# Data-driven linearization of dynamical systems

**DOI:** 10.1007/s11071-024-10026-x

**Published:** 2024-08-15

**Authors:** George Haller, Bálint Kaszás

**Affiliations:** Institute for Mechanical Systems, ETH Zürich, Leonhardstrasse 21, 8092 Zurich, Switzerland

## Abstract

Dynamic mode decomposition (DMD) and its variants, such as extended DMD (EDMD), are broadly used to fit simple linear models to dynamical systems known from observable data. As DMD methods work well in several situations but perform poorly in others, a clarification of the assumptions under which DMD is applicable is desirable. Upon closer inspection, existing interpretations of DMD methods based on the Koopman operator are not quite satisfactory: they justify DMD under assumptions that hold only with probability zero for generic observables. Here, we give a justification for DMD as a local, leading-order reduced model for the dominant system dynamics under conditions that hold with probability one for generic observables and non-degenerate observational data. We achieve this for autonomous and for periodically forced systems of finite or infinite dimensions by constructing linearizing transformations for their dominant dynamics within attracting slow spectral submanifolds (SSMs). Our arguments also lead to a new algorithm, data-driven linearization (DDL), which is a higher-order, systematic linearization of the observable dynamics within slow SSMs. We show by examples how DDL outperforms DMD and EDMD on numerical and experimental data.

**Supplementary Information:**

The online version contains supplementary material available at 10.1007/s11071-024-10026-x.

## Introduction

In recent years, there has been an overwhelming interest in devising linear models for dynamical systems from experimental or numerical data (see the recent review by Schmid [51]). This trend was largely started by the dynamic mode decomposition (DMD), put forward in seminal work by Schmid [50]. The original exposition of the method has been streamlined by various authors, most notably by Rowley et al. [49] and Kutz et al. [32].

To describe DMD, we consider an autonomous dynamical system

1 $$\begin{aligned} \dot{\textbf{x}}=\textbf{f}(\textbf{x}),\qquad \textbf{x}\in {\mathbb {R}}^{n},\qquad \textbf{f}\in C^{1}\left( {\mathbb {R}}^{n}\right) , \end{aligned}$$

for some $n\in \mathbb {N}^{+}$. Trajectories $\left\{ \textbf{x}(t;\textbf{x}_{0})\right\} _{t\in \mathbb {R}}$ of this system evolve from initial conditions $\textbf{x}_{0}$. The flow map $\textbf{F}^{t}:{\mathbb {R}}^{n}\rightarrow {\mathbb {R}}^{n}$ is defined as the mapping taking the initial trajectory positions at time $t_{0}=0$ to current ones at time *t*, i.e.,

2 $$\begin{aligned} \textbf{F}^{t}(\textbf{x}_{0})=\textbf{x}(t;\textbf{x}_{0}). \end{aligned}$$

As observations of the full state space variable $\textbf{x}$ of system (1) are often not available, one may try to explore the dynamical system (1) by observing *d* smooth scalar functions $\phi _{1}(x),\ldots ,{{\phi }}_{d}(x)$ along trajectories of the system. We order these scalar observables into the observable vector

3 $$\begin{aligned} \varvec{\phi }(x)=\left( \begin{array}{c} \phi _{1}(\textbf{x})\\ \vdots \\ \phi _{d}(\textbf{x}) \end{array}\right) \in C^{1}\left( {\mathbb {R}}^{n}\right) \end{aligned}$$

The basic idea of DMD is to approximate the observed evolution of $\varvec{\phi }\left( \textbf{F}^{t}(\textbf{x}_{0})\right)$ of the dynamical system with the closest fitting autonomous linear dynamical system

4 $$\begin{aligned} \dot{\varvec{\phi }}=\textbf{L}\varvec{\phi },\qquad \textbf{L}\in \mathbb {R}^{d\times d}, \end{aligned}$$

based on available trajectory observations.

This is a challenging objective for multiple reasons. First, the original dynamical system (1) is generally nonlinear whose dynamics cannot be well approximated by a single linear system on a sizable open domain. For instance, one may have several isolated, coexisting attracting or repelling stationary states (such as periodic orbits, limit cycles or quasiperiodic tori), which linear systems cannot have. Second, it is unclear why the dynamics of *d* observables should be governed by a self-contained autonomous dynamical system induced by the original system (1), whose dimension is *n*. Third, the result of fitting system (4) to observable data will clearly depend on the initial conditions used, the number and the functional form of the observables chosen, as well as on the objective function used in minimizing the fit.

Despite these challenges, we may proceed to find an appropriately defined closest linear system (4) based on available observable data. We assume that for some fixed time step $\Delta t$, discrete observations of *m* initial conditions, $\textbf{x}^{1}(t_{0}),\ldots ,\textbf{x}^{m}(t_{0})$, and their images $\textbf{F}^{\Delta t}(\textbf{x}^{1}(t_{0})),\ldots ,\textbf{F}^{\Delta t}\left( \textbf{x}^{m}(t_{0})\right)$, under the sampled flow map $\textbf{F}^{\Delta t}$ are available in the data matrices

5 $$\begin{aligned} \varvec{\Phi }= & {} \left[ \varvec{\phi }\left( \textbf{x}^{1}(t_{0})\right) ,\ldots ,\varvec{\phi }\left( \textbf{x}^{m}(t_{0})\right) \right] ,\qquad \nonumber \\ \hat{\varvec{\Phi }}= & {} \left[ \varvec{\phi }\left( \textbf{F}^{\Delta t}(\textbf{x}^{1}(t_{0}))\right) ,\ldots ,\varvec{\phi }\left( \textbf{F}^{\Delta t}\left( \textbf{x}^{m}(t_{0})\right) \right) \right] ,\nonumber \\ \end{aligned}$$

respectively. We seek the best fitting linear system of the form (4) for which

6 $$\begin{aligned} \hat{\varvec{\Phi }}\approx \varvec{\mathcal {D}}\varvec{\Phi },\quad \varvec{\mathcal {D}}=e^{\textbf{L}\Delta t}, \end{aligned}$$

holds. The eigenvalues of such a $\varvec{\mathcal {D}}$ are usually called DMD eigenvalues, and their corresponding eigenvectors are called the DMD modes.

Various norms can be chosen with respect to which the difference of $\hat{\varvec{\Phi }}$ and ${\varvec{\mathcal {D}}}\varvec{\Phi }$ is to be minimized. The most straightforward choice is the Euclidean matrix norm $\left| \,\cdot \,\right|$, which leads to the minimization principle

7 $$\begin{aligned} \varvec{\mathcal {D}}^{*}=\underset{\varvec{\mathcal {D}}\in \mathbb {R}^{d\times d}}{\textrm{argmin}\left| \hat{\varvec{\Phi }}-\varvec{\mathcal {D}}\varvec{\Phi }\right| ^{2}}. \end{aligned}$$

An explicit solution to this problem is given by

8 $$\begin{aligned} \varvec{\mathcal {D}}=\left( \hat{\varvec{\Phi }}\varvec{\Phi }^{\textrm{T}}\right) \left( \hat{\varvec{\Phi }}\varvec{\Phi }^{\textrm{T}}\right) ^{\dagger }, \end{aligned}$$

with the dagger referring to the pseudo-inverse of a matrix (see, e.g., Kutz et al. [32] for details). We note that the original formulation of Schmid [50] is for discrete dynamical processes and assumes observations of a single trajectory (see also Rowley et al. [49]).

Among several later variants of DMD surveyed by Schmid [51], the most broadly used one is the Extended Dynamic Mode Decomposition (EDMD) of Williams et al. [58]. This procedure seeks the best-fitting linear dynamics for an a priori unknown set of functions $\textbf{K}(\varvec{\phi }(\textbf{x}))$ of $\varvec{\phi }(\textbf{x})$, rather than for $\varvec{\phi }(\textbf{x})$ itself. In practice, one often chooses $\textbf{K}$ as an *N*(*d*, *k*)-dimensional vector of *d*-variate scalar monomials of order *k* or less, where $$N(d,k)=\left( \begin{array}{c} d+k\\ k \end{array}\right) $$ is the total number of all such monomials. The underlying assumption of EDMD is that a self-contained linear dynamical system of the form

9 $$\begin{aligned} \frac{d}{dt}\textbf{K}\left( \varvec{\phi }(\textbf{F}^{t}(\textbf{x}_{0}))\right) =\textbf{L}\textbf{K}\left( \varvec{\phi }(\textbf{F}^{t}(\textbf{x}_{0}))\right) \end{aligned}$$

can be obtained on the feature space $\mathbb {R}^{N(d,k)}$ by optimally selecting $\textbf{L}\in \mathbb {R}^{N(d,k)\times N(d,k)}$. For physical systems, the *N*(*d*, *k*)-dimensional ODE in Eq. (9) defined on the feature space $\mathbb {R}^{N(d,k)}$ can be substantially higher-dimensional than the *d*-dimensional ODE (4). In fact, *N*(*d*, *k*) may be substantially higher than the dimension *n* of the phase space $\mathbb {R}^{n}$ of the original nonlinear system (1).

Once the function library used in EDMD is fixed, one again seeks to choose $\textbf{L}$ so that

$$\begin{aligned} \textbf{K}(\hat{\varvec{\Phi }})\approx \varvec{\mathcal {D}}\textbf{K}(\varvec{\Phi }),\quad \varvec{\mathcal {D}}=e^{\textbf{L}\Delta t}. \end{aligned}$$

This again leads to a linear optimization problem that can be solved using linear algebra tools. For higher-dimensional systems, a kernel-based version of EDMD was developed by Williams et al. [59]. This method computes inner products necessary for EDMD implicitly, without requiring an explicit representation of (polynomial) basis functions in the space of observables. As a result, kernel-based EDMD operates at computational costs comparable to those of the original DMD.

## Prior justifications for DMD methods

Available justifications for DMD (see [49]) and EDMD (see [58]) are based on the Koopman operator, whose basics we review in Appendix A.1 for completeness. The argument starts with the observation that special observables falling in invariant subspaces of this operator in the space of all observables obey linear dynamics. Consequently, DMD should recover the Koopman operator restricted to this subspace if the observables are taken from such a subspace.

In this sense, DMD is viewed as an approximate, continuous immersion of a nonlinear system into an infinite dimensional linear dynamical system. While such an immersion is not possible for typical nonlinear systems with multiple limit sets (see [38, 39]), one still hopes that this approximate immersion is attainable via DMD or EDMD for nonlinear systems with a single attracting steady state that satisfies appropriate nondegeneracy conditions (see [33]). In that case, unlike classic local linearization near fixed points, the linearization via DMD or EDMD is argued to be non-local, as it covers the full domain of definition of Koopman eigenfunctions spanning the underlying Koopman-invariant subspace.

However, Koopman eigenfunctions, whose existence, domain of definition and exact form are a priori unknown for general systems, are notoriously difficult–if not impossible–to determine accurately from data. More importantly, even if Koopman-invariant subspaces of the observable space were known, any countable set of generically chosen observables would lie outside those subspaces with probability one. As a consequence, DMD eigenvectors (which are generally argued to be approximations of Koopman eigenfunctions and can be used to compute Koopman modes^1^) would also lie outside Koopman-invariant subspaces, given that such eigenvectors are just linear combinations of the available observables. Consequently, practically observed data sets would fall under the realm of Koopman-based explanation for DMD with probability zero. This is equally true for EDMD, whose flexibility in choosing the function set $\textbf{K}(\varvec{\phi }(\textbf{x}))$ of observables also introduces further user-dependent heuristics beyond the dimension *d* of the DMD.

One may still hope that by enlarging the dimension *d* of observables in DMD and enlarging the function library $\textbf{K}(\varvec{\phi }(\textbf{x}))$ in EDMD, the optimization involved in these methods brings DMD and EDMD eigenvectors closer and closer to Koopman eigenfunctions. The required enlargements, however, may mean hundreds or thousand of dimensions even for dynamical system governed by simple, low-dimensional ODEs [59]. These enlargements succeed in fitting linear systems closely to sets of observer trajectories, but they also unavoidably lead to overfits that give unreliable predictions for initial conditions not used in the training of DMD or EDMD. Indeed, the resulting large linear systems can perform substantially worse in prediction than much lower dimensional linear or nonlinear models obtained from other data-driven techniques (see, e.g., Alora et al. [1]).

Similar issues arise in justifying the kernel-based EDMD of Williams et al. [59] based on the Koopman operator. Additionally, the choice of the kernel function that represents the inner product of the now implicitly defined polynomial basis functions remains heuristic and problem-dependent. Again, the accuracy of the procedure is not guaranteed, as available observer data is generically not in a Koopman eigenspace. As Williams et al. [59] write: “Like most existing data-driven methods, there is no guarantee that the kernel approach will produce accurate approximations of even the leading eigenvalues and modes, but it often appears to produce useful sets of modes in practice if the kernel and truncation level of the pseudoinverse are chosen properly.”

Finally, a lesser known limitation of the Koopman-based approach to DMD is the limited domain in the phase space over which Koopman eigenfunctions (and hence their corresponding invariant subspaces) are defined in the observable space. Specifically, at least one principal Koopman eigenfunction necessarily blows up near basin boundaries of attracting and repelling fixed points and periodic orbits (see Proposition 1 of our Appendix A.4 for a precise statement and Theorem 3 of Kvalheim and Arathoon [33] for a more general related result).

Expansions of observables in terms of such blowing-up eigenfunctions have even smaller domains of convergence, as was shown explicitly in a simple example by Page and Kerswell [44]. This is a fundamental obstruction to the often envisioned concept of global linear models built of different Koopman eigenfunctions over multiple domains of attraction (see, e.g., Williams et al. [58], p. 1309). While it is broadly known that such models would be discontinuous along basin boundaries [33, 38, 39], it is rarely noted (see Kvalheim and Arathoon [33] for a rare exception) that such models would also generally blow up at those boundaries and hence would become unmanageable even before reaching the boundaries.

For these reasons, an alternative mathematical foundation for DMD is desirable. Ideally, such an approach should be defined on an equal or lower dimensional space, rather than on higher or even infinite-dimensional spaces, as suggested by the Koopman-based view on DMD. This should help in avoiding overfitting and computational difficulties. Additionally, an ideal treatment of DMD should also provide specific non-degeneracy conditions on the underlying dynamical system, on the available observables, and on the specific data to be used in the DMD procedure.

In this paper, we develop a treatment of DMD that satisfies these requirements. This enables to derive conditions for DMD to approximate the dominant linearized observable dynamics near hyperbolic fixed points and periodic orbits of finite- and infinite-dimensional dynamical systems.

Our approach to DMD also leads to a refinement of DMD which we call data-driven linearization (or DDL, for short). DDL effectively carries out exact local linearization via nonlinear coordinate changes on a lower-dimensional attracting invariant manifold (spectral submanifold) of the dynamical system. We illustrate the increased accuracy and domain of validity of DDL models relative to those obtained from DMD and EDMD on examples of autonomous and forced dynamical systems.

## A simple justification for the DMD algorithm

Here we give an alternative interpretation of DMD and EDMD as approximate models for a dynamical system known through a set of observables. The main idea (to be made more precise shortly) is to view DMD executed on *d* observables $\phi _{1},\ldots ,\phi _{d}$ as a model reduction tool that captures the leading-order dynamics of $n\ge d$ phase space variables along a *d*-dimensional slow manifold in terms of $\phi _{1},\ldots ,\phi _{d}$.

**Fig. 1:**
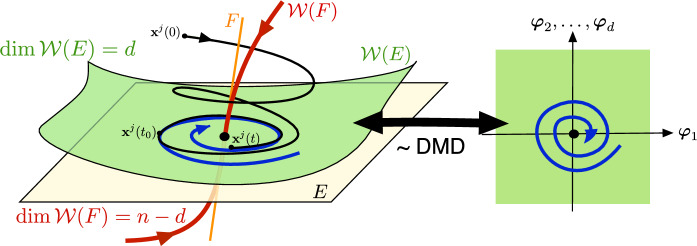
The geometric meaning of DMD performed on *d* observables, $\phi _{1}(\textbf{x}),\ldots ,\phi _{d}(\textbf{x})$, after initial fast transients die out at an exponential rate in the data. DMD then identifies the leading-order (linear) dynamics on a *d*-dimensional attracting spectral submanifold (SSM) $\mathcal {W}\left( E\right)$ tangent to the *d*-dimensional slow spectral subspace *E*. These linearized dynamics can be expressed in terms of the SSM-restricted observables $\varvec{\varphi }=\varvec{\phi }\vert _{\mathcal {W}\left( E\right) }$. Also shown is the spectral subspace *F* of the faster decaying modes and its associated nonlinear continuation, the fast spectral subspace $\mathcal {W}\left( F\right)$

Such manifolds arise as slow spectral submanifolds (SSMs) under weak non-degeneracy assumptions on the linearized spectrum at stable hyperbolic fixed points of the *n*-dimensional dynamical system (see [10, 12, 22]). Specifically, a slow SSM $\mathcal {W}\left( E\right)$ is tangent to the real eigenspace *E* spanned by the *d* slowest decaying linearized modes at the fixed point. If *m* sample trajectories $\left\{ \textbf{x}^{j}(t)\right\} _{j=1}^{m}$ are released from a set of initial conditions $\left\{ \textbf{x}^{j}(0)\right\} _{j=1}^{m}$at time $t=0$, then due to their fast decay along the remaining fast spectral subspace *F* , the $\textbf{x}^{j}(t)$ trajectories will become exponentially close to $\mathcal {W}\left( E\right)$ by some time $t=t_{0}\ge 0$, and closely synchronize with its internal dynamics, as seen in Fig. 1.

If $\mathcal {W}\left( E\right)$ admits a non-degenerate parametrization with respect to the observables $\phi _{1},\ldots ,\phi _{d}$, then one can pass to these observables as new coordinates in which to approximate the leading order, linearized dynamics inside $\mathcal {W}\left( E\right)$. Specifically, DMD executed over time the interval $\left[ t_{0},t_{0}+\Delta t\right]$ provides the closest linear fit to the reduced dynamics of $\mathcal {W}\left( E\right)$ from the available observable histories $\phi _{1}\left( \textbf{x}^{j}(t)\right) ,\ldots ,\phi _{d}\left( \textbf{x}^{j}(t)\right)$ for $t\in \left[ t_{0},t_{0}+\Delta t\right]$, as illustrated in Fig. 1. This fit is a close representation of the actual linearized dynamics on the SSM $\mathcal {W}\left( E\right)$ if the trajectory data $\left\{ \textbf{x}^{j}(t)\right\} _{j=1}^{m}$ is sufficiently diverse for $t\ge t_{0}$. The resulting DMD model with coefficient matrix ${\varvec{\mathcal {D}}}$ will then be smoothly conjugate to the linearized reduced dynamics on $\mathcal {W}\left( E\right)$ with an error of the order of the distance of$\left\{ \textbf{x}^{j}(t)\right\} _{j=1}^{m}$ from $\mathcal {W}\left( E\right)$ between the times $t_{0}$ and $t_{0}+\Delta t$.

The slow SSM $\mathcal {W}\left( E\right)$ may also contain unstable modes in applications. Similar results hold for that case as well, provided that we select a small enough $\Delta t$, ensuring that the trajectories $\left\{ \textbf{x}^{j}(0)\right\} _{j=1}^{m}$are not ejected from the vicinity of the fixed point origin for $t\in \left[ t_{0},t_{0}+\Delta t\right]$. As DMD will show sensitivity with respect to the choice of $t_{0}$ and $\Delta t$ in this case, we will not discuss the justification of DMD near unstable fixed points beyond Remarks 1 and 7.

In the following sections, we make this basic idea more precise both for finite and infinite-dimensional dynamical systems. This approach also reveals explicit, previously undocumented non-degeneracy conditions on the underlying dynamical system, on the available observable functions and on the specific data set used, under which DMD should give meaningful results.

### Justification of DMD for continuous dynamical systems

We start by assuming that the observed dynamics take place in a domain containing a fixed point, which is assumed to be at the origin, without loss of generality, i.e.,

10 $$\begin{aligned} \textbf{f}(\textbf{0})=\textbf{0}. \end{aligned}$$

We can then rewrite the dynamical system (1) in the more specific form

11 $$\begin{aligned} \dot{\textbf{x}}= & {} \textbf{Ax}+\tilde{\textbf{f}}(\textbf{x}),\qquad \textbf{x}\in {\mathbb {R}}^{n},\qquad \textbf{A}=D\textbf{f}(\textbf{0}),\qquad \nonumber \\ \tilde{\textbf{f}}(\textbf{x})= & {} o\left( \left| \textbf{x}\right| \right) , \end{aligned}$$

where the classic $o\left( \left| \textbf{x}\right| \right)$ notation refers to the fact that $\lim _{\textbf{x}\rightarrow \textbf{0}}\left[ \left| \tilde{\textbf{f}}(\textbf{x})\right| /\left| \textbf{x}\right| \right] =0$.

Most expositions of DMD methods and their variants do not state assumption (10) explicitly and hence may appear less restrictive than our treatment here. However, all of them implicitly assume the existence of such a fixed point, as all of them end up returning homogeneous linear ODEs or mappings with a fixed point at the origin. Indeed, all known applications of these methods that produce reasonable accuracy target the dynamics of ODEs or discrete maps near their fixed points.

Assumption (10) can be replaced with the existence of a limit cycle in the original system (1), in which case the first return map (or Poincaré map) defined near the limit cycle will have a fixed point. We give a separate treatment on justifying DMD as a linearization for such a Poincaré map in Sect. 3.2.

We do not advocate, however, the often used procedure of applying DMD to fit a linear system to the flow map (rather than the Poincaré map) near a stable limit cycle. Such a fit only produces the desired limiting periodic behavior if one or more of the DMD eigenvalues are artificially constrained to be on the complex unit circle by the user of DMD. This renders the DMD model both structurally unstable and conceptually inaccurate for prediction. Indeed, the model will approximate the originally observed limit cycle and convergence to it only within a measure zero, cylindrical set of its phase space. Outside this set, all trajectories of the DMD model will converge to some other member of the infinite family of periodic orbits or invariant tori within the center subspace corresponding to the unitary eigenvalues. These periodic orbits or tori have a continuous range of locations and amplitudes, and hence represent spurious asymptotic behaviors that are not seen in the original dynamical system (1).

In the special case of $d=n$ and for the special observable $\varvec{\Phi } (\textbf{x})= \textbf{x}$, the near-linear form of Eq. (11) motivates the DMD procedure because a linear approximation to the system near $\textbf{x}=\textbf{0}$ seems feasible. It is a priori unclear, however, to what extent the nonlinearities distort the linear dynamics and how DMD would account for that. Additionally, in a data-driven analysis, choosing the full phase space variable $\textbf{x}$ as the observable $\varvec{\phi }(\textbf{x})$ is generally unrealistic. For these reasons, a mathematical justification of DMD requires further assumptions, as we discuss next.

Let $\lambda _{1},\ldots ,\lambda _{n}\in \mathbb {C}$ denote the eigenvalues of $\textbf{A}$ and let $\textbf{e}_{1},\ldots ,\textbf{e}_{n}\in \mathbb {C}^{n}$ denote the corresponding generalized eigenvectors. We assume that at least one of the modes of the linearized system at $\textbf{x}=\textbf{0}$ decays exponentially and there is at least one other mode that decays slower or even grows. More specifically, for some positive integer $d<n$, we assume that the spectrum of *A* can be partitioned as

12 $$\begin{aligned} \textrm{Re}{{\lambda _{n}}}\le \ldots \le \textrm{Re}{{\lambda _{d+1}} }<\textrm{Re}{{\lambda _{d}} }\le \ldots \le \textrm{Re}{{\lambda _{1}<0}.}\nonumber \\ \end{aligned}$$

This guarantees the existence of a *d*-dimensional, normally attracting* slow spectral subspace*

13 $$\begin{aligned} E=\textrm{span}\,\left\{ \textrm{Re}\,{{\textbf{e}_{1}},\,}\textrm{Im}\,{{\textbf{e}_{1}},}\ldots ,\textrm{Re}\,{{\textbf{e}_{d}},\,}\textrm{Im}\,{{\textbf{e}_{d}}}\right\} \end{aligned}$$

for the linearized dynamics, with linear decay rate towards *E* strictly dominating all decay rates inside *E*. Note that the set of vectors $\textrm{Re}\, \textbf{e}_1, \textrm{Im}\, \textbf{e}_1, \ldots , \textrm{Re}\, \textbf{e}_{d}, \textrm{Im}\, \textbf{e}_{d}$ is, in general, not linearly independent, but they span a *d*-dimensional subspace. We also define the (real) spectral subspace of faster decaying linear modes:

14 $$\begin{aligned} F=\textrm{span}\,\left\{ \textrm{Re}\,{{\textbf{e}_{d+1}},\,}\textrm{Im}\,{{\textbf{e}_{d+1}},}\ldots ,\textrm{Re}\,{{\textbf{e}_{n}},\,}\textrm{Im}\,{{\textbf{e}_{n}}}\right\} . \end{aligned}$$

We will also use matrices containing the left and right eigenvectors of the operator $\textbf{A}$ and of its restrictions, $\textbf{A}\vert _{E}$ and $\textbf{A}\vert _{F}$, to its spectral subspaces *E* and *F*, respectively. Specifically, we let

15 $$\begin{aligned} \textbf{T}&=\left[ \textbf{T}_{E},\textbf{T}_{F}\right] ,\quad \textbf{P}=\left[ \begin{array}{c} \textbf{P}_{E}\\ \textbf{P}_{F} \end{array}\right] , \end{aligned}$$

where the columns of $\textbf{T}_{E}\in \mathbb {R}^{n\times d}$ are the real and imaginary parts of the generalized right eigenvectors of $\textbf{A}\vert _{E}$ and the columns of $\textbf{T}_{F}\in \mathbb {R}^{n\times (n-d)}$ are defined analogously for $\textbf{A}\vert _{F}.$ Similarly, the rows of $\textbf{P}_E\in \mathbb {R}^{d\times n}$ are the real and imaginary parts of the generalized left eigenvectors of $\textbf{A}\vert _{E}$ and the rows of $\textbf{P}_{F}\in \mathbb {R}^{(n-d)\times n}$ are defined analogously for$\textbf{A}\vert _{F}.$ Under assumption (12), *F* is always a fast spectral subspace, containing all trajectories of the linearized system that decay faster to the origin as any trajectory in *E*.

We will use the notation

16 $$\begin{aligned} \textbf{X}= & {} \left[ \textbf{x}^{1}(t_{0}),\ldots ,\textbf{x}^{m}(t_{0})\right] ,\quad \nonumber \\ \hat{\textbf{X}}= & {} \left[ \textbf{F}^{\Delta t}(\textbf{x}^{1}(t_{0})),\ldots ,\textbf{F}^{\Delta t}\left( \textbf{x}^{m}(t_{0})\right) \right] \end{aligned}$$

for trajectory data in the underlying dynamical system (1) on which the observable data matrices $\varvec{\Phi }$ and $\hat{\varvec{\Phi }}$ defined in (5) are defined. In truly data-driven applications, the matrices $\textbf{X}$ and $\hat{\textbf{X}}$ are not known. We will nevertheless use them to make precise statements about a required dominance of the slow linear modes of *E* in the available data. Such a dominance will arise for generic initial conditions if one selects the initial conditions $\textbf{x}^{1}(t_{0}),\ldots ,\textbf{x}^{m}(t_{0})$ after initial fast transients along *F* have died out. This can be practically achieved by initializing $\textbf{x}^{1}(t_{0}),\ldots ,\textbf{x}^{m}(t_{0})$ after a linear spectral analysis of the observable data matrix $\varvec{\Phi }$ returns a number of dominant frequencies consistent with a *d*-dimensional SSM.

We now state a theorem that provides a general justification for the DMD procedure under explicit nondegeneracy conditions and with specific error bounds. Specifically, we give a minimal set of conditions under which DMD can be justified as an approximate, leading-order, *d*-dimensional reduced-order model for an nonlinear system of dimension $n\ge d$ near its fixed point. Based on relevance for applications, we only state Theorem 1 for stable hyperbolic fixed points, but discuss subsequently in Remark 1 its extension to unstable fixed points.

#### Theorem 1

(Justification of DMD for ODEs with stable hyperbolic fixed points) Assume that

1. The origin, $\textbf{x}=0$, is a stable hyperbolic fixed point of system (11) with a spectral gap, i.e., the spectrum $\textrm{Spect}\left[ \textbf{A}\right]$ satisfies Eq. (12).
2. $\textbf{f}\in C^{2}$ in a neighborhood of the origin.
3. For some integer $d\in \left[ 1,n\right]$, a *d*-dimensional observable function $\varvec{\phi }\in C^{2}$ and the *d*-dimensional slow spectral subspace *E* of the hyperbolic fixed point $\textbf{x}=\textbf{0}$ of system (11) satisfy the non-degeneracy condition
  17 $$\begin{aligned} \textrm{rank}\,\left[ D\varvec{\phi }\left( \textbf{0}\right) \vert _{E}\right] =d. \end{aligned}$$
4. The data matrices $\varvec{\Phi }$ and $\hat{\varvec{\Phi }}$ are non-degenerate and the initial conditions in $\textbf{X}$ and $\hat{\textbf{X}}$ have been selected after fast transients from the modes outside *E* have largely died out, i.e.,
  18 $$\begin{aligned} \textrm{rank}_{\textrm{row}}\,\varvec{\Phi }=d,\qquad \left| \textbf{P}_{F}\textbf{X}\right| ,\left| \textbf{P}_{F}\hat{\textbf{X}}\right| \le \left| \textbf{P}_{E}\textbf{X}\right| ^{1+\beta },\nonumber \\ \end{aligned}$$
  for some $$\beta \in (0,1]$$.

Then the DMD computed from $\varvec{\Phi }$ and $\hat{\varvec{\Phi }}$ yields a matrix $\varvec{\mathcal {D}}$ that is locally topologically conjugate with order $\mathcal {O}\left( \left| \textbf{P}_{E}\textbf{X}\right| ^{\beta }\right)$ error to the linearized dynamics on a *d*-dimensional, slow attracting spectral submanifold $\mathcal {W}(E)\in C^{1}$ tangent to *E* at $\textbf{x}=\textbf{0}$. Specifically, we have

19 $$\begin{aligned} {\varvec{\mathcal {D}}}=D\varvec{\phi }(\textbf{0})\textbf{T}_{E}e^{{\varvec{\Lambda }}_{E}\Delta t} \left( D\varvec{\phi }(\textbf{0})\textbf{T}_{E}\right) ^{-1}+\mathcal {O}\left( \left| \textbf{P}_{E}\textbf{X}\right| ^{\beta }\right) .\nonumber \\ \end{aligned}$$

#### Proof

Under assumptions (A1) and (A2), any trajectory in a neighborhood of the origin in the nonlinear system (11) converges at an exponential rate $e^{\textrm{Re}{{\lambda _{d+1}} }t}$ to a *d*-dimensional attracting spectral submanifold $\mathcal {W}(E)$ tangent to a *d*-dimensional attracting slow spectral subspace *E* of the linearized system at the origin. This follows from the $C^{1}$ linearization theorem of Hartman [23], which is applicable to $C^{2}$ dynamical systems with a stable hyperbolic fixed point. Under assumption (A3), the *d*-dimensional observable function $\varvec{\phi }(\textbf{x})$ restricted to $\mathcal {W}(E)$ can be used to parametrize $\mathcal {W}(E)$ near the origin, and hence a *d*-dimensional, self-contained nonlinear dynamical system can be written down for the restricted observable $\varvec{\varphi }=\varvec{\phi }\vert _{\mathcal {W}(E)}$ along $\mathcal {W}(E)$. Under the first assumption in (A4), the available observational data matrices $\varvec{\Phi }$ and $\hat{\varvec{\Phi }}$ are rich enough to characterize the reduced dynamics on $\mathcal {W}(E)$. Under the second assumption in (A4), transients from the faster modes outside *E* have largely died out before the selection of the initial conditions in $\textbf{X}$, so that the linear part of the dynamics on $\mathcal {W}(E)$ can be approximately inferred from $\varvec{\Phi }$ and $\hat{\varvec{\Phi }}$. In that case, up to an error proportional to the distance of the training data from $\mathcal {W}(E)$, the matrix $\varvec{\mathcal {D}}\in \mathbb {R}^{d\times d}$ returned by DMD is similar to the time-$\Delta t$ flow map of the linearized flow of the underlying dynamical system restricted to $\mathcal {W}(E)$. This linearized flow then acts as a local reduced-order model with which nearby trajectory observations synchronize exponentially fast in the observable space. We give a more detailed proof of the theorem in Appendix (B). $\square$

#### Remark 1

In Theorem 1, we can replace assumption (A1) with

20 $$\begin{aligned}{} & {} \text {Re} \lambda _n \le ... \le \text {Re} \lambda _{d+1}<0,\nonumber \\{} & {} \text {Re} \lambda _{d+1} < \text {Re} \lambda _{d} \le ... \le \text {Re} \lambda _1, \nonumber \\{} & {} \text {Re}\lambda _{j} \ne 0, j = 1, ..., n\nonumber \\ \end{aligned}$$

This means that the $\textbf{x}=\textbf{0}$ fixed point is only assumed hyperbolic with a spectral gap and $\textbf{A}$ has an attracting *d*-dimensional spectral subspace *E* that possibly contains some instabilities, i.e., eigenvalues with positive real parts. Then the statements of Theorem 1 still hold, but $\mathcal {W}(E)$ will be only be guaranteed $C^{1}$ at $\textbf{x}=\textbf{0}$ and Hölder-continuous at other points near the fixed point. This follows by replacing the linearization theorem of Hartman [23] with that of van Strien [56], which still enables us to use Eq. (79) in the proof. Therefore, slow subspaces *E* containing a mixture of stable and unstable modes can also be allowed, as long as *F* contains only fast modes consistent with the splitting assume in Eq. (12). In that case, however, the time $t_{0}+\Delta t$ must be chosen carefully to ensure that $\left| \textbf{P}_{F}\hat{\textbf{X}}\right| \le \left| \textbf{P}_{E}\textbf{X}\right| ^{1+\beta }$ still holds, i.e., the data used in DMD still samples a neighborhood of the origin.

#### Remark 2

In related work, Bollt et al. [7] construct the transformation relating a pair of conjugate dynamical systems based on a limited set of matching Koopman eigenfunctions, which are either known explicitly or constructed from EDMD with dictionary learning (EDMD-DL; see [36]). In principle, this could be used to construct linearizing transformation as well. However, even when the eigenfunctions are approximated from data, the approach assumes that the linearized system, as well as a linearized trajectory and its preimage under the linearization, are available. As these assumptions are not satisfied in practice, only very simple and low-dimensional analytic examples are treated by Bollt et al. [7].

In Appendix (B), Remarks 4 and 5 summarize technical points on the application and possible further extensions of Theorem 1. In practice, Theorem 1 provides previously unspecified non-degeneracy conditions on the linear part of the dynamical system to be analyzed via DMD (assumption (A1)), on the regularity of the nonlinear part of the system (assumption (A2)), on the type of observables available for the analysis (assumption (A3)) and on the specific observable data used in the analysis (assumption (A4)). The latter assumption requires that there have to be at least as many independent observations in time as observables. This specifically excludes the popular use of tall $\varvec{\Phi }$ observable data matrices which provide more free parameters to pattern-match observational data but will also lead to an overfit that diminishes the predictive power of the DMD model on initial conditions not used in its training.

To illustrate these points, we demonstrate the necessity of assumptions (A2)–(A4) of Theorem 1 in Appendix C on simple examples.

### Justification of DMD for discrete and for time-periodic continuous dynamical systems

The linearization results we have applied to deduce Theorem 1 are equally valid for discrete dynamical systems defined by iterated mappings. Such mappings are of the form

21 $$\begin{aligned} \textbf{x}_{n+1}= & {} \textbf{f}(\textbf{x}_{n})=\textbf{A}\textbf{x}_{n}+\tilde{\textbf{f}}(\textbf{x}_{n}),\qquad \textbf{x}_{j}\in \mathbb {R}^{n},\qquad \nonumber \\{} & {} \textbf{A}\in \mathbb {R}^{n\times n},\qquad \tilde{\textbf{f}}(\textbf{x})=o\left( \left| \textbf{x}\right| \right) . \end{aligned}$$

We will use a similar ordering for the eigenvalues of $\textbf{A}$ as in the continuous time case:

22 $$\begin{aligned} \left| {{\lambda _{n}}}\right| \le \ldots \le \left| {{\lambda _{d+1}} }\right|<\left| {{\lambda _{d}} }\right| \le \ldots \le \left| {{\lambda _{1}} }\right| <1. \end{aligned}$$

As in the continuous time case, we will use the observable data matrices

23 $$\begin{aligned} \varvec{\Phi }=\varvec{\phi }\left( \textbf{X}\right) ,\qquad \hat{\varvec{\Phi }}=\varvec{\phi }\left( \textbf{f}\left( \textbf{X}\right) \right) , \end{aligned}$$

with the initial conditions for the map $\textbf{f}$ stored in $\textbf{X}$.

With these ingredients, we need only minor modifications in the assumptions of the theorems that account for the usual differences between the spectrum of an ODE and a map.

#### Theorem 2

(Justification of DMD for maps with stable hyperbolic fixed points) Assume that

1. $\textbf{x}=\textbf{0}$ is a stable hyperbolic fixed point of system (21), i.e., assumption (22) holds.
2. In Eq. (21), $\tilde{\textbf{f}}\in C^{2}$ holds in a neighborhood of the origin.
3. For some integer $d\in \left[ 1,n\right]$, a *d*-dimensional observable function $\varvec{\phi }\in C^{2}$ and the *d*-dimensional slow spectral subspace *E* of the hyperbolic fixed point $\textbf{x}=\textbf{0}$ of system (21) satisfy the non-degeneracy condition
  24 $$\begin{aligned} \textrm{rank}\,\left[ D\varvec{\phi }\left( \textbf{0}\right) \vert _{E}\right] =d. \end{aligned}$$
4. The data matrices $\varvec{\Phi }$ and $\hat{\varvec{\Phi }}$ collected from iterations of system (21) are non-degenerate and are dominated by data near *E*, i.e.,
  25 $$\begin{aligned}{} & {} \textrm{rank}_{\textrm{row}}\,\varvec{\Phi }=d,\nonumber \\{} & {} \left| \textbf{P}_{F}\textbf{X}\right| ,\left| \textbf{P}_{F}\hat{\textbf{X}}\right| \le \left| \textbf{P}_{E}\textbf{X}\right| ^{1+\beta }, \end{aligned}$$
  for some $$\beta \in (0,1)$$.

Then the DMD computed from $\varvec{\Phi }$ and $\hat{\varvec{\Phi }}$ yields a matrix $\varvec{\mathcal {D}}$ that is locally topologically conjugate with order $\mathcal {O}\left( \left| \textbf{P}_{E}\textbf{X}\right| ^{\beta }\right)$ error to the linearized dynamics on a *d*-dimensional attracting spectral submanifold $\mathcal {W}(E)\in C^{1}$ tangent to *E* at $\textbf{x}=\textbf{0}$. Specifically, we have

26 $$\begin{aligned} {\varvec{\mathcal {D}}}=D\varvec{\phi }(\textbf{0})\textbf{T}_{E}{\varvec{\Lambda }}_{E}\left( D\varvec{\phi }(\textbf{0})\textbf{T}_{E}\right) ^{-1}+\mathcal {O} \left( \left| \textbf{P}_{E}\textbf{X}\right| ^{\beta }\right) . \end{aligned}$$

The spectral submanifold $\mathcal {W}(E)$ and its reduced dynamics are of class $C^{1}$ at the origin, and at least Hölder continuous in a neighborhood of the origin.

#### Proof

The proof is identical to the proof of Theorem 1 but uses the discrete version of the linearization result by Hartman [23] for stable hyperbolic fixed points of maps.

Theorem 1 can be immediately applied to justify DMD as a linearization tool for period-one maps (or Poincaré maps) of time-periodic, non-autonomous dynamical systems near their periodic orbits. This requires the data matrices $\varvec{\Phi }$ and $\hat{\varvec{\Phi }}$ to contain trajectories of such a Poincaré map. Remark 8 on the treatment of slow spectral subspaces *E* containing possible instabilities also applies here under the modified assumption

27 $$\begin{aligned}{} & {} \left| {{\lambda _{n}}}\right| \le \ldots \le \left| {{\lambda _{d+1}} }\right|<1,\nonumber \\{} & {} \left| {{\lambda _{d+1}} }\right| <\left| {{\lambda _{d}} }\right| \le \ldots \le \left| {{\lambda _{1}} }\right| ,\nonumber \\{} & {} \left| {{\lambda _{j}} }\right| \ne 1, j=1,\ldots ,n, \end{aligned}$$

which only requires the fixed point to be hyperbolic and $\textbf{A}$ to have a *d*-dimensional normally attracting subspace.

### Justification of DMD for infinite-dimensional dynamical systems

Most data sets of interest arguably arise from infinite-dimensional dynamical systems of fluids and solids. Examples include experimental or numerical data describing fluid motion, continuum vibrations, climate dynamics or salinity distribution in the ocean. In the absence of external forcing, these problems are governed by systems of autonomous nonlinear partial differential equations that can often be viewed as evolutionary differential equations in a form similar to Eq. (1), but defined on an appropriate infinite-dimensional Banach space. Accordingly, time-sampled solutions of these equations can be viewed as iterated mappings of the form (21) but defined on Banach spaces.

Our approach to justifying DMD generally carries over to this infinite-dimensional setting, as long as the observable vector $\varvec{\phi }(\textbf{x})$ remains finite-dimensional, and both the Banach space and the discrete or continuous dynamical system defined on it satisfy appropriate regularity conditions. These regularity conditions tend to be technical, but when they are satisfied, they do guarantee the extension of Theorems 1 and 2 to Banach spaces. This offers a justification to use DMD to obtain an approximate finite-dimensional linear model for the dynamics of the underlying continuum system on a finite-dimensional attracting slow manifold (or inertial manifold) in the neighborhood of a non-degenerate stationary solution.

To avoid major technicalities, we only state here a generalized version of Theorem 1 to justify the use of DMD for observables defined on Banach spaces for a discrete evolutionary process with a stable hyperbolic stationary state. We consider mappings of the form

28 $$\begin{aligned} \textbf{x}_{n+1}= & {} \textbf{f}(\textbf{x}_{n})=\textbf{A}\textbf{x}_{n}+\tilde{\textbf{f}}(\textbf{x}_{n}),\qquad \textbf{x}_{j}\in \mathcal {B},\qquad \nonumber \\{} & {} \tilde{\textbf{f}}:U\subset \mathcal {B}\rightarrow \mathcal {B},\qquad \tilde{\textbf{f}}(\textbf{0})=\textbf{0}\in U, \end{aligned}$$

where $\mathcal {B}$ is a Banach space, *U* is an open set in $\mathcal {B}$, and $\textbf{A}:\mathcal {B}\rightarrow \mathcal {B}$ is an invertible linear operator that is bounded in the norm defined on $\mathcal {B}$. The function $\textbf{f}$ can be here the time-sampled version of an infinite-dimensional flow map of an autonomous evolutionary PDE or the Poincaré map of a time-periodic evolutionary PDE. We assume that for some $\alpha \in \left( 0,1\right)$, $\tilde{\textbf{f}}\in C^{1,\alpha }(U)$ holds, i.e., $\tilde{\textbf{f}}$ is (Fréchet-) differentiable in *U* and its derivative, $D\tilde{\textbf{f}}$, is Hölder-continuous in $\textbf{x}\in U$ with Hölder exponent $\alpha$.

The *spectral radius* of *A* is defined as

$$\begin{aligned} \rho (\textbf{A})=\lim _{k\rightarrow \infty }\left| \textbf{A}^{k}\right| ^{\frac{1}{k}}. \end{aligned}$$

We recall that in the special case $\mathcal {B}=\mathbb {R}^{n}$ treated in Sect. 3.2, we have $\rho (\textbf{A})=\max _{1\le j\le n}\left| {{\lambda _{j}}}\right|$. For some $\alpha \in \left( 0,1\right)$, the linear operator $\textbf{A}$ is called $\alpha$-*contracting* if

29 $$\begin{aligned} \rho (\textbf{A})^{1+\alpha }\rho \left( \textbf{A}^{-1}\right) <1, \end{aligned}$$

which can only hold if $\rho (\textbf{A})<1$ (see [41]). Therefore, in the simple case of $\mathcal {B}=\mathbb {R}^{n}$, *A* is $\alpha$-*contracting* if it is a contraction (i.e., all its eigenvalues are less than one in norm) and

$$\begin{aligned} \left| \lambda _{1}\right| ^{1+\alpha }<\left| \lambda _{n}\right| , \end{aligned}$$

showing that the spectrum of $\textbf{A}$ is confined to an annulus of outer radius $\left| \lambda _{1}\right| <1$ and inner radius $\left| \lambda _{1}\right| ^{1+\alpha }$. We can now state our main result on the justification of DMD for infinite-dimensional discrete dynamical systems.

#### Theorem 3

(Justification of DMD for infinite-dimensional maps with stable hyperbolic fixed points) Assume that

1. For some $\alpha \in \left( 0,1\right)$, the linear operator $\textbf{A}$ is $\alpha$-contracting (and hence the $x=0$ fixed point of system (28) is linearly stable).
2. In Eq. (28), $\tilde{\textbf{f}}\in C^{1,\alpha }(U)$ holds in a *U* neighborhood of the origin.
3. For some integer $d\in \mathbb {N}^{+}$, there is a splitting $\mathcal {B}=E\oplus F$ of $\mathcal {B}$ into two *A*-invariant subspaces $E,F\subset \mathcal {B}$ such that *E* is *d*-dimensional and slow, i.e.,
  30 $$\begin{aligned} \rho \left( \textbf{A}\vert _{E}\right) <\frac{1}{\rho \left( \textbf{A}^{-1}\vert _{F}\right) } \end{aligned}$$
  Furthermore, a *d*-dimensional observable function $\varvec{\phi }\in C^{2}$ satisfies the non-degeneracy condition
  31 $$\begin{aligned} \textrm{rank}\,\left[ D\varvec{\phi }\left( \textbf{0}\right) \vert _{E}\right] =d. \end{aligned}$$
4. The data matrices $\varvec{\Phi }$ and $\hat{\varvec{\Phi }}$ collected from iterations of system (28) are non-degenerate and are dominated by data near *E*, i.e.,
  32 $$\begin{aligned}{} & {} \textrm{rank}_{\textrm{row}}\,\varvec{\Phi }=d,\nonumber \\{} & {} \left| \textbf{P}_{F}\textbf{X}\right| ,\left| \textbf{P}_{F}\hat{\textbf{X}}\right| \le \left| \textbf{P}_{E}\textbf{X}\right| ^{1+\beta }, \end{aligned}$$
  for some $$\beta \in (0,1)$$.

Then the DMD computed from $\varvec{\Phi }$ and $\hat{\varvec{\Phi }}$ yields a matrix $\varvec{\mathcal {D}}$ that is locally topologically conjugate with order $\mathcal {O}\left( \left| \textbf{P}_{E}\textbf{X}\right| ^{\beta }\right)$ error to the linearized dynamics on a *d*-dimensional attracting spectral submanifold $\mathcal {W}(E)$ tangent to *E* at $\textbf{x}=\textbf{0}$. Specifically, we have

33 $$\begin{aligned} \varvec{\mathcal {D}}=D\varvec{\phi }(\textbf{0})\textbf{T}_{E}{\varvec{\Lambda }}_{E}\left( D\varvec{\phi }(\textbf{0})\textbf{T}_{E}\right) ^{-1}+\mathcal {O}\left( \left| \textbf{P}_{E}\textbf{X}\right| ^{\beta }\right) . \end{aligned}$$

The spectral submanifold $\mathcal {W}(E)$ and its reduced dynamics are of class $C^{1}$ in a neighborhood of the origin.

#### Proof

The proof follows the steps in the proof of Theorem 2 but uses an infinite-dimensional linearization result, Theorem 3.1 of Newhouse [41], for stable hyperbolic fixed points of maps on Banach spaces. Specifically, if $\textbf{A}$ is $\alpha$-contracting, then Newhouse [41] shows the existence of a near-identity linearizing transformation $\textbf{x}=\textbf{y}+\textbf{h}(\textbf{y})$ for the discrete dynamical system (28) such that $\textbf{h}\in C^{1,\alpha }(B)$ holds on a small enough ball $B\subset U$ centered at $\textbf{x}=\textbf{0}$. Using this linearization theorem instead of its finite-dimensional version from Hartman [23], we can follow the same steps as in the proof of Theorem 2 to conclude the statement of the theorem. $\square$

In Appendix B, Remarks 6 and 7 summarize technical remarks on possible further extensions of Theorem 3.

## Data-driven linearization (DDL)

### Theoretical foundation for DDL

Based on the results of the previous section, we now refine the first-order approximation to the linearized dynamics yielded by DMD near a hyperbolic fixed point. Specifically, we construct the specific nonlinear coordinate change that linearizes the restricted dynamics on the attracting spectral submanifold $\mathcal {W}(E)$ illustrated in Fig. 1. This classic notion of linearization on $\mathcal {W}(E)$ yields a *d*-dimensional linear reduced model, which can be of significantly lower dimension than the original *n*-dimensional nonlinear system. This is to be contrasted with the broadly pursued Koopman embedding approach (see, e.g., [8, 40, 49]), which seeks to immerse nonlinear systems into linear systems of dimensions substantially higher (or even infinite) relative to *n*.

The following result gives the theoretical basis for our subsequent data-driven linearization (DDL) algorithm. We will use the notation $C^{a}$ for the class of real analytic functions. We also use the notation $\left\lfloor {x}\right\rfloor$ to denote the integer part of *x*.

#### Theorem 4

(DDL principle for ODEs with a stable hyperbolic fixed points) Assume that the origin, $\textbf{x}=\textbf{0}$ is a stable hyperbolic fixed point of system (11) and the spectrum of $\textbf{A}$ has a spectral gap as in Eq. (12). Assume further that for some $r\in \mathbb {N}^{+}\cup \left\{ \infty ,a\right\}$, the following conditions are satisfied:

1. $\textbf{f}_{2}\in C^{r}$ and the nonresonance conditions
  34 $$\begin{aligned} \lambda _{k}\ne & {} \sum _{j=1}^{n}m_{j}\lambda _{j},\qquad m_j \in \mathbb {N}, k = 1, ..., n,\qquad \nonumber \\ 2\le & {} \sum _{j=1}^{n}m_{j}\le Q\le r,\quad \nonumber \\ Q:= & {} \left\lfloor {\frac{\max _{i}\left| \textrm{Re}\,\lambda _{i}\right| }{\min _{i}\left| \textrm{Re}\,\lambda _{i}\right| }}\right\rfloor +1, \end{aligned}$$
  hold for the eigenvalues of $\textbf{A}$.
2. For some integer $d\in \left[ 1,n\right]$, a *d*-dimensional observable function $\varvec{\phi }\in C^{r}$ and the *d*-dimensional slow spectral subspace *E* of the stable fixed point $\textbf{x}=\textbf{0}$ of system (11) satisfy the non-degeneracy condition.
  35 $$\begin{aligned} \textrm{rank}\,\left[ D\varvec{\phi }\left( \textbf{0}\right) \vert _{E}\right] =d. \end{aligned}$$

Then the following hold:

(i) On the unique *d*-dimensional attracting spectral submanifold $\mathcal {W}(E)\in C^{r}$ tangent to *E* at $x=0$, the reduced observable vector $\varvec{\varphi }=\varvec{\phi }\vert _{\mathcal {W}(E)}$ can be used to describe the reduced dynamics as
  36 $$\begin{aligned} \dot{\varvec{\varphi }}= & {} \textbf{B}\varvec{\varphi }+\textbf{q}\left( \varvec{\varphi }\right) ,\qquad \nonumber \\ \textbf{B}= & {} D\varvec{\phi }(\textbf{0})\textbf{T}_{E}\varvec{\Lambda }_{E}\left( D\varvec{\phi }(\textbf{0})\textbf{T}_{E}\right) ^{-1},\quad \nonumber \\ \textbf{q}\left( \varvec{\varphi }\right)= & {} \mathcal {O}\left( \left| \varvec{\varphi }\right| ^{2}\right) . \end{aligned}$$
(ii) There exists a unique, $C^{r}$ change of coordinates
  37 $$\begin{aligned} \varvec{\varphi }=\varvec{\kappa }(\varvec{\gamma })=\varvec{\gamma }+\varvec{\ell }(\varvec{\gamma }), \end{aligned}$$
  that transforms the reduced dynamics on $\mathcal {W}(E)$ to its linearization
  38 $$\begin{aligned} \dot{\varvec{\gamma }}=\textbf{B}\varvec{\gamma } \end{aligned}$$
  inside the domain of attraction of $\textbf{x}=\textbf{0}$ within the spectral submanifold $\mathcal {W}(E).$
(iii) The transformation (37) satisfies the *d*-dimensional system of nonlinear PDEs
  39 $$\begin{aligned} D_{\varvec{\gamma }}\varvec{\ell }(\varvec{\gamma })\textbf{B}\varvec{\gamma }=\textbf{B}\varvec{\ell }(\varvec{\gamma })+\textbf{q}\left( \varvec{\gamma }+\varvec{\ell }(\varvec{\gamma })\right) . \end{aligned}$$
  If $r\in \mathbb {N}^{+}\cup \left\{ \infty \right\}$, solutions of this PDE can locally be approximated as
  40 $$\begin{aligned} \varvec{\ell }(\varvec{\gamma })\!= & {} \!\sum _{\left| \textbf{k}\right| \!=\!2}^{r}\textbf{l}_{\textbf{k}}\varvec{\gamma }^{\textbf{k}}\!+\!o\left( \left| \varvec{\gamma }\right| ^{r}\right) ,\,\textbf{k}\in \mathbb {N}^{d},\,\textbf{l}_{\textbf{k}}\in \mathbb {R}^{d},\quad \nonumber \\ \varvec{\gamma }^{\textbf{k}}:= & {} \gamma _{1}^{k_{1}}\cdots \gamma _{d}^{k_{d}}. \end{aligned}$$
  If $r=a$, then the local approximation (40) can be refined to a convergent Taylor series
  41 $$\begin{aligned} \varvec{\ell }(\varvec{\gamma })= & {} \sum _{\left| \textbf{k}\right| =2}^{\infty }\textbf{l}_{\textbf{k}}\varvec{\gamma }^{\textbf{k}},\quad \textbf{k}\in \mathbb {N}^{d},\quad \textbf{l}_{\textbf{k}}\in \mathbb {R}^{d},\quad \nonumber \\ \varvec{\gamma }^{\textbf{k}}:= & {} \gamma _{1}^{k_{1}}\cdots \gamma _{d}^{k_{d}} \end{aligned}$$
  in a neighborhood of the origin. In either case, the coefficients $\textbf{l}_{\textbf{k}}$ can be determined by substituting the expansion for $\varvec{\ell }(\varvec{\gamma })$ into the PDE (39), equating coefficients of equal monomials $\varvec{\gamma }^{\textbf{k}}$ and solving the corresponding recursive sequence of *d*-dimensional linear algebraic equations for increasing $\left| \textbf{k}\right|$.

#### Proof

The proof builds on the existence of the *d*-dimensional spectral submanifold $\mathcal {W}\left( E\right)$ guaranteed by Theorem 1. For a $C^{r}$ dynamical system with $r\in \mathbb {N}^{+}\cup \left\{ \infty ,a\right\}$, $\mathcal {W}\left( E\right)$ is also $C^{r}$ smooth based on the linearization theorems of Poincaré [46] and Sternberg [52], as long as the nonresonance condition (34) holds. Condition (35) then ensures that $\mathcal {W}\left( E\right)$ can be parametrized locally by the restricted observable vector $\varvec{\varphi }$ and hence its reduced dynamics can be written as a nonlinear ODE for $\varvec{\varphi }$. This ODE can again be linearized by a near-identity coordinate change (37) using the appropriate linearization theorem of the two cited above. The result is the restricted linear system (38) to which the dynamics is $C^{r}$ conjugate within the whole domain of attraction of the $\varvec{\varphi }=\textbf{0}$ fixed point inside $\mathcal {W}\left( E\right)$. The invariance PDE (39) can be obtained by substituting the linearizing transformation (37) into the reduced dynamics on $\mathcal {W}\left( E\right)$. This PDE can then be solved via a Taylor expansion up to order *r*. We give more a more detailed proof in Appendix D. $\square$

Note that 1:1 resonances are not excluded by the condition (34), and hence repeated eigenvalues arising from symmetries in physical systems are still amenable to DDL. Also of note is that the non-resonance conditions (34) do not exclude frequency-type resonances among imaginary parts of oscillatory eigenvalues. Rather, they exclude simultaneous resonances of the same type between the real and the imaginary parts of the eigenvalues. Such resonances will be absent in data generated by generic oscillatory systems.

Assuming hyperbolicity is essential for Theorem 4 to hold, since in this case the linearization is the same as transforming the dynamics to the Poincaré-normal form. For a non-hyperbolic fixed point, this normal form transformation results in nonlinear dynamics on the center manifold. This would, however, only arise in highly non-generic systems, precisely tuned to be at criticality. Since this is unlikely to happen in experimentally observed or numerically simulated systems, the hyperbolicity assumption is not restrictive.

Finally, under the conditions of Theorem 3, the DDL results of Theorem 4 also apply to data from infinite-dimensional dynamical systems, such as the fluid sloshing experiments we will analyze using DDL in Sect. 5.4. In practice, the most restrictive condition of Theorem 3 is (A1), which requires the solution operator to have a spectrum uniformly bounded away from zero. Such uniform boundedness is formally violated in important classes of infinite-dimensional evolution equations, presenting a technical challenge for the direct applications of SSM results to certain delay-differential equations (see [54]) and partial differential equations (see, e.g., [9, 30]). However, this challenge only concerns rigorous conclusions on the existence and smoothness of a finite-dimensional, attracting SSM. If the existence of such an SSM is convincingly established from an alternative mathematical theory (as is [9]) or inferred from data (as in [54]), then the DDL algorithm based on Theorem 4 can be used to obtain a data-driven linearization of the dynamics on that SSM.

### DDL versus EDMD

Here we examine whether there is a possible relationship between DDL and the extended DMD (or EDMD) algorithm of Williams et al. [58]. For simplicity, we assume analyticity for the dynamical system ($r=a$) and hence we can write the inverse of the linearizing transformation (40) behind the DDL algorithm as a convergent Taylor expansion of the form

42 $$\begin{aligned} \varvec{\gamma }=\varvec{\kappa }^{-1}\left( \varvec{\varphi }\right) =\varvec{\varphi }+\sum _{\left| \textbf{k}\right| =2}^{\infty }\textbf{q}_{\textbf{k}}\varvec{\varphi }^{\textbf{k}}. \end{aligned}$$

We then differentiate this equation in time to obtain from the linearized equation (38) a *d*-dimensional system of equations

$$\begin{aligned} \dot{\varvec{\varphi }}+\sum _{\left| \textbf{k}\right| =2}^{\infty }\textbf{q}_{\textbf{k}}\frac{d}{dt}\varvec{\varphi }^{\textbf{k}}=\textbf{B}\varvec{\varphi }+\sum _{\left| \textbf{k}\right| =2}^{\infty }\textbf{B}\textbf{q}_{\textbf{k}}\varvec{\varphi }^{\textbf{k}} \end{aligned}$$

that the restricted observable $\varvec{\varphi }$ and its monomials $\varvec{\varphi }^{\textbf{k}}$ must satisfy. This last equation can be rewritten as a *d*-dimensional autonomous system of linear system of ODEs,

43 $$\begin{aligned} \left[ \begin{array}{cc} \textbf{I}_{d\times d}&\textbf{Q}_{2}\end{array}\right] \frac{d}{dt}\left[ \begin{array}{c} \varvec{\varphi }\\ \textbf{K}_{\ge 2}\left( \varvec{\varphi }\right) \end{array}\right] \!=\!\left[ \begin{array}{cc} \textbf{B}\,\,&\textbf{B}\textbf{Q}_{2}\end{array}\right] \left[ \begin{array}{c} \varvec{\varphi }\\ \varvec{K}_{\ge 2}\left( \varvec{\varphi }\right) \end{array}\right] , \nonumber \\ \end{aligned}$$

for the reduced observable $\varvec{\varphi }$ and the infinite-dimensional vector $\textbf{K}_{\ge 2}(\varvec{\varphi })$ of all nonlinear monomials of $\varvec{\varphi }$. Here $\textbf{I}_{d\times d}$ denotes the *d*-dimensional identity matrix and $\textbf{Q}_{2}$ contains all coefficients $\textbf{q}_{\textbf{k}}$ as column vectors starting from order $\left| \textbf{k}\right| =2$.

If we truncate the infinite-dimensional vector of monomials $\textbf{K}_{\ge 2}\left( \varvec{\varphi }\right)$ to the vector $\textbf{K}_{2}^{k}\left( \varvec{\varphi }\right)$ of nonlinear monomials up to order *k*, then Eq. (43) becomes

44 $$\begin{aligned} \left[ \begin{array}{cc} \textbf{I}_{d\times d}&\textbf{Q}_{2}^{k}\end{array}\right] \frac{d}{dt}\left[ \begin{array}{c} \varvec{\varphi }\\ \textbf{K}_{2}^{k}\left( \varvec{\varphi }\right) \end{array}\right] \!=\!\left[ \begin{array}{cc} \textbf{B}&\textbf{B}\textbf{Q}_{2}^{k}\end{array}\right] \left[ \begin{array}{c} \varvec{\varphi }\\ \textbf{K}_{2}^{k}\left( \varvec{\varphi }\right) \end{array}\right] . \nonumber \\ \end{aligned}$$

This is a *d*-dimensional implicit system of linear ODEs for the dependent variable vector $\left( \varvec{\varphi },\textbf{K}_{2}^{k}\left( \varvec{\varphi }\right) \right)$ whose dimension is always larger than *d*. Consequently, the operator $$\left[ \begin{array}{cc} \textbf{I}_{d\times d}&\textbf{Q}_{2}^{k}\end{array}\right] $$ is never invertible and hence, contrary to the assumption of EDMD, there is no well-defined linear system of ODEs that governs the evolution of an observable vector and the monomials of its components.

The above conclusion remains unchanged even if one attempts to optimize with respect to the choice of the coefficients $\textbf{q}_{\textbf{k}}$ in the matrix $\textbf{Q}_{2}^{k}$.

### Implementation and applications of DDL

#### Basic implementation of DDL for model reduction and linearization

Theorem 4 allows us to define a numerical procedure to construct a linearizing transformation on the *d*-dimensional attracting slow manifold $\mathcal {W}(E)$ systematically from data. From Eq. (44), the matrices $\textbf{B}$ and $\textbf{Q}$ are to be determined, given a set of observed trajectories. In line with the notation used in Sect. 4.2, let the data matrix $\textbf{K}_{2}^{k}\left( \varvec{\varphi }\right)$ contain monomials (from order 2 to order *k*) of the observable vector $\varvec{\varphi }$ and let $\hat{\textbf{K}_{2}^{k}}\left( \varvec{\varphi }\right)$ contain denote the evaluation of $\textbf{K}_{2}^{k}\left( \varvec{\varphi }\right)$ time $\Delta t$ later. Passing to the discrete version of the invariance equation (44), we obtain

$$\begin{aligned} \left[ \begin{array}{cc} \textbf{I}&\textbf{Q}\end{array}\right] \left[ \begin{array}{c} \hat{\varvec{\varphi }}\\ \hat{\textbf{K}_{2}^{k}}\left( \varvec{\varphi }\right) \end{array}\right] =\left[ \begin{array}{cc} \varvec{\mathcal {B}}&\varvec{\mathcal {B}}\textbf{Q}\end{array}\right] \left[ \begin{array}{c} \varvec{\varphi }\\ \textbf{K}_{2}^{k}\left( \varvec{\varphi }\right) \end{array}\right] \end{aligned}$$

for some matrices $\textbf{Q}\in \mathbb {R}^{d\times N(d,k)-d}$ and $\varvec{\mathcal {B}}=e^{\textbf{B}\Delta t}\in \mathbb {R}^{d\times d}$. Moreover, the inverse transformation of the linearization on the SSM $\mathcal {W}\left( E\right)$ is well-defined, and hence with an appropriate matrix $\textbf{Q}^{inv}\in \mathbb {R}^{d\times N(d,k)-d}$, we can write

$$\begin{aligned} \left[ \begin{array}{cc} \textbf{I}&\textbf{Q}^{inv}\end{array}\right] \left[ \begin{array}{c} \varvec{\varphi }+\textbf{Q}\textbf{K}_{2}^{k}\left( \varvec{\varphi }\right) \\ \textbf{K}_{2}^{k}\left( \varvec{\varphi }+\textbf{Q}\textbf{K}_{2}^{k}\left( \varvec{\varphi }\right) \right) \end{array}\right] =\varvec{\varphi }. \end{aligned}$$

This allows us to define the cost functions

45 $$\begin{aligned} \mathcal {L}^{(1)}(\textbf{Q},\varvec{\mathcal {B}})&=\left| \left[ \begin{array}{cc} \textbf{I}&\textbf{Q}\end{array}\right] \left[ \begin{array}{c} \hat{\varvec{\varphi }}\\ \hat{\textbf{K}_{2}^{k}}\left( \varvec{\varphi }\right) \end{array}\right] \right. \nonumber \\&\quad \left. -\left[ \begin{array}{cc} \varvec{\mathcal {B}}&\varvec{\mathcal {B}}\textbf{Q}\end{array}\right] \left[ \begin{array}{c} \varvec{\varphi }\\ \textbf{K}_{2}^{k}\left( \varvec{\varphi }\right) \end{array}\right] \right| ^{2},\nonumber \\ \mathcal {L}^{(2)}\left( \textbf{Q},\textbf{Q}^{inv}\right)&=\left| \textbf{Q}\textbf{K}_{2}^{k}\left( \varvec{\varphi }\right) \right. \nonumber \\&\quad \left. +\textbf{Q}^{inv}\textbf{K}_{2}^{k}\left( \varvec{\varphi }+\textbf{Q}\textbf{K}_{2}^{k}\left( \varvec{\varphi }\right) \right) \right| ^{2}, \end{aligned}$$

where $\mathcal {L}^{(1)}$ measures the invariance error along the observed trajectories and $\mathcal {L}^{(2)}$ measures the error due to the computation of the inverse. We aim to jointly minimize $\mathcal {L}^{(1)}$ and $\mathcal {L}^{(2)}$. To this end, we define the combined cost function

46 $$\begin{aligned} \mathcal {L}_{\nu }\left( \textbf{Q},\textbf{Q}^{inv},\varvec{\mathcal {B}}\right)= & {} \mathcal {L}^{(1)}(\textbf{Q},\varvec{\mathcal {B}})\nonumber \\{} & {} +\nu \mathcal {L}^{(2)}\left( \textbf{Q},\textbf{Q}^{inv}\right) , \end{aligned}$$

for some $\nu \ge 0$. In our examples, we choose $\nu =1$, which puts the same weight on both terms in the cost function (46). Minimizers of $\mathcal {L}_{\nu }$ provide optimal solutions to the DDL principle and can be written as

47 $$\begin{aligned} (\textbf{Q}^{\star },\textbf{Q}^{inv,\star }\varvec{\mathcal {B}}^{\star })=\underset{\textbf{Q},\textbf{Q}^{inv},\varvec{\mathcal {B}}}{\textrm{argmin}}\,\,\mathcal {L_{\nu }}(\textbf{Q},\textbf{Q}^{inv},\varvec{\mathcal {B}}), \end{aligned}$$

or, equivalently, as solutions of the system of equations

48 $$\begin{aligned} \frac{\partial \mathcal {L}_{\nu }}{\partial Q_{ij}}&=0\quad i=1,...,d,j=1,...,N(d,k)-d, \end{aligned}$$

49 $$\begin{aligned} \frac{\partial \mathcal {L}_{\nu }}{\partial Q_{ij}^{inv}}&=0,\quad i=1,...,d,j=1,...,N(d,k)-d,\nonumber \\ \frac{\partial \mathcal {L}_{\nu }}{\partial \mathcal {B}_{ij}}&=0\quad i,j=1,...,d. \end{aligned}$$

The optimal solution (47) does not necessarily coincide with the Taylor-coefficients of the linearizing transformation (41). Instead of giving the best local approximation, $(\textbf{Q}^{\star },\textbf{Q}^{inv,\star }\varvec{\mathcal {B}}^{\star })$ approximates the linearizing transformation and the linear dynamics in a least-squares sense over the domain of the training data. This means that DDL is not hindered by the convergence properties of the analytic linearization. Note that for $d=1$, one can estimate the radius of convergence of (41), for example, by constructing the Domb–Skyes plot (see [15]), or by finding the radius of the circle in the complex plane onto which the roots of the truncated expansion accumulate under increasing orders of truncation (see [25, 47]). For $d>1$, such analysis is more difficult, since multivariate Taylor-series have more complicated domains of convergence. In our numerical examples, we estimate the domain of convergence of such analytic linearizations as the domain on which $\varvec{\kappa }\circ \varvec{\kappa }^{-1}=\textbf{I}$ holds to a good approximation. As we will see, this domain of convergence may be substantially smaller the domain of validity of transformations determined in a fully data-driven way.

Since the cost function (45) is not convex, the optimization problem (47) has to be solved iteratively starting from an initial guess $(\textbf{Q}_{0},\textbf{Q}_{0}^{inv},\varvec{\mathcal {B}}_{0})$. For the examples presented in the paper, we use the Levenberg–Marquardt algorithm (see [4]), but other nonlinear optimization methods, such as gradient descent or Adam (see [29]) could also be used. For our implementation, which is available from the repository [28], we used the Scipy and Pytorch libraries of Virtanen et al. [57], Paszke et al. [45]. In summary, we will use the following Algorithm 1 in our examples for model reduction via DDL.

**Algorithm 1:**
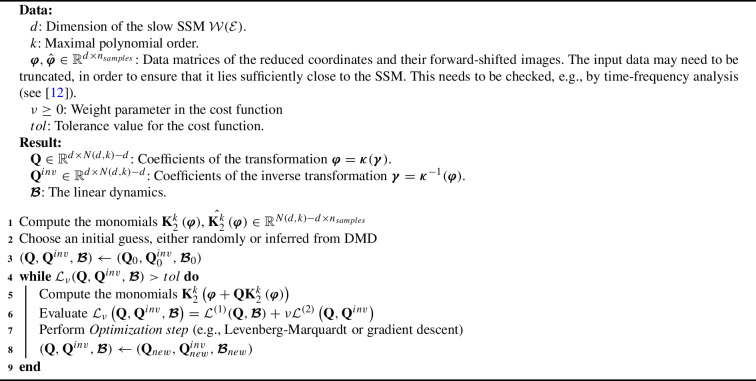
Model reduction with DDL

##### Remark 3

The expressions (45)–(46) define one of the possible choices for the cost function. With $\nu =0$, (46) simply corresponds to a one-step-ahead prediction with the linearized dynamics. Alternatively, a multi-step prediction can also be enforced. For a training trajectory $\varvec{\varphi }(t),$ the invariance

$$\begin{aligned} \left[ \begin{array}{cc} \textbf{I}&\textbf{Q}\end{array}\right] \left[ \begin{array}{c} \varvec{\varphi }\\ \textbf{K}_{2}^{k}\left( \varvec{\varphi }\right) \end{array}\right] =\left[ \begin{array}{cc} \varvec{\mathcal {B}}^{1:m}&\varvec{\mathcal {B}}^{1:m}\textbf{Q}\end{array}\right] \left[ \begin{array}{c} \varvec{\varphi }({0})\\ \textbf{K}_{2}^{k}\left( \varvec{\varphi }({0})\right) \end{array}\right] \end{aligned}$$

could be required, where $\varvec{\mathcal {B}}^{1:m}$ is a tensor composed of powers of the linear map $\varvec{\mathcal {B}}$. Optimizing over the entire trajectory is, however, more costly than simply minimizing (45), and we found no noticeable improvement in accuracy in our numerical examples.

#### Relationship with DMD implementations

Note that setting $\textbf{Q}=\textbf{Q}^{inv}=\textbf{0}$ in the optimization problem (47) turns the problem into DMD. In this case, the usual DMD algorithm surveyed in the Introduction returns

$$\begin{aligned} {\varvec{\mathcal {B}}}_{0}=\underset{\varvec{\mathcal {B}}}{\textrm{argmin}}\,\,{\mathcal {L}_{0}(\textbf{0},\textbf{0},\varvec{\mathcal {B}})}, \end{aligned}$$

which is a good initial guess for the non-convex optimization problem (47). More importantly, since Theorem 4 guarantees the existence of a near-identity linearizing transformation, we expect that the true minimizer is close to the DMD-solution. Therefore, we may explicitly expand the cost function (45) around the DMD solution as

$$\begin{aligned}&\mathcal {L_{\nu }}(\textbf{Q},\textbf{Q}^{inv},{\varvec{\mathcal {B}}}) =\mathcal {L}_{\nu }(\textbf{0},\textbf{0},\varvec{\mathcal {B}}_{0})\\&\quad +D\mathcal {L}_{(\textbf{0},\textbf{0}, {\varvec{\mathcal {B}}}_{0})}\cdot \left( \begin{array}{c} \textbf{Q}\\ \textbf{Q}^{inv}\\ {\varvec{\mathcal {B}}}-\varvec{\mathcal {B}}_{0} \end{array}\right) \\&\quad +\frac{1}{2}\left[ D^{2}\mathcal {L}_{(\textbf{0},\textbf{0},{\varvec{\mathcal {B}}}_{0})}\cdot \left( \begin{array}{c} \textbf{Q}\\ \textbf{Q}^{inv}\\ \varvec{\mathcal {B}}-\varvec{\mathcal {B}}_{0} \end{array}\right) \right] \cdot \left( \begin{array}{c} \textbf{Q}\\ \textbf{Q}^{inv}\\ \varvec{\mathcal {B}}-\varvec{\mathcal {B}}_{0} \end{array}\right) \\&\quad +\mathcal {}\left( \left| \textbf{Q}\right| ^{3},\left| \textbf{Q}^{inv}\right| ^{3},\left| \varvec{\mathcal {B}}-\varvec{\mathcal {B}}_{0}\right| ^{3}\right) , \end{aligned}$$

where $D\mathcal {L}_{(\textbf{0},\textbf{0},\varvec{\mathcal {B}}_{0})}$ and $D^{2}\mathcal {L}_{(\textbf{0},\textbf{0},\varvec{\mathcal {B}}_{0})}$ are the Jacobian and the Hessian of the cost function evaluated at the DMD solution, respectively. Since the Jacobian is nonsingular at the DMD solution, the minimum of the quadratic approximation of the cost function satisfies the linear equation

50 $$\begin{aligned} -D\mathcal {L}_{(\textbf{0},\textbf{0},{\varvec{\mathcal {B}}}_{0})}=D^{2}\mathcal {L}_{(\textbf{0},\textbf{0},\varvec{\mathcal {B}}_{0})}\left( \begin{array}{c} \textbf{Q}\\ \textbf{Q}^{inv}\\ \varvec{\mathcal {B}}-\varvec{\mathcal {B}}_{0} \end{array}\right) . \end{aligned}$$

This serves as the first-order correction to the DMD-solution in the DDL procedure. The Eq. (50) is explicitly solvable and is equivalent to performing a single Levenberg–Marquardt step on the non-convex cost function (45), with the DMD solution $(\textbf{0},\textbf{0},{\varvec{\mathcal {B}}}_{0})$ serving as an initial guess.

Minimization of (45) leads to a non-convex optimization problem. Besides computing the leading-order approximation (50), a possible workaround to this challenge is to carry out the linearization in two steps. First, one can fit a polynomial map to the reduced dynamics by linear regression. Then, if the reduced-dynamics is non-resonant, it can be analytically linearized. Axås et al. [3] follow this approach to automatically find the extended normal form style reduced dynamics on SSMs using the implementation of SSM Tool by Jain et al. [24]. Although this procedure does convert the DDL principle into a convex problem, the drawback is that the linearization is obtained as a Taylor-expansion, with possibly limited convergence properties.

#### Using DDL to construct spectral foliations

The mathematical foundation of SSM-reduced modeling is that any trajectory converging to a slow SSM is guaranteed to synchronize up to an exponentially decaying error with one of the trajectories on the SSM. This follows from the general theory of invariant foliations by Fenichel [18], when applied to the *d*-dimensional normally hyperbolic invariant manifold $\mathcal {W}(E)$.^2^ The main result of the theory is that off-SSM initial conditions synchronizing with the same on-SSM trajectory turn out to form a class $C^{r-1}$ smooth, $\left( n-d\right)$-dimensional manifold, denoted $\mathcal {F}_{\textbf{p}}$, which intersects $\mathcal {W}(E)$ in a unique point $\textbf{p}\in \mathcal {W}(E)$. The manifold $\mathcal {F}_{\textbf{p}}$ is called the stable fiber emanating from the base point $\textbf{p}$. Fenichel proves that any off-SSM trajectory $\textbf{x}(t;\textbf{x}_{0})$ with initial condition $\textbf{x}_{0}\in {{\mathcal {F}_{\textbf{p}_{0}}} }$ converges to the specific on-SSM trajectory $\textbf{p}(t;\textbf{p}_{0})\in \mathcal {W}(E)$ with initial condition $\textbf{p}_{0}\in \mathcal {W}(E)$ faster than any other nearby trajectory might converge to $\textbf{p}(t;\textbf{p}_{0}).$ Recently, Szalai [55] studied this foliation in more detail under the name “invariant spectral foliation”, discussed its uniqueness in an appropriate smoothness class and proposed its use in model reduction.

To predict the evolution of a specific, off-SSM initial condition $\textbf{x}_{0}$ up to time *t* from an SSM-based model, we first need to relate that initial condition to the base point $\textbf{p}_{0}$ of the stable fiber $\mathcal {F}_{\textbf{p}_{0}}$. Next, we need to run the SSM-based reduced model up to time *t* to obtain $\textbf{p}(t;\textbf{p}_{0})$. Based on the exponentially fast convergence of the full solution $\textbf{x}(t;\textbf{x}_{0})$ to the SSM-reduced solution $\textbf{p}(t;\textbf{p}_{0}),$ we obtain an accurate longer-term prediction for $\textbf{x}(t;\textbf{x}_{0})$ using this procedure. Such a longer-term prediction is helpful, for instance, when we wish to predict steady states, such as fixed points and limit cycles, from the SSM-reduced dynamics.

Constructing this spectral foliation directly from data, however, is challenging for nonlinear systems. Indeed, one would need a very large number of initial conditions that cover uniformly a whole open neighborhood of the fixed point in the phase space. For example, while one or two training trajectories are generally sufficient to infer accurate SSM-reduced models even for very high-dimensional systems (see e.g., [3, 12, 13]), thousands of uniformly distributed initial conditions in a whole open set of a fixed point are required to infer accurate spectral foliation-based models even for low-dimensional systems (see [55]). The latter number and distribution of initial conditions is unrealistic to acquire in a truly data-driven setting.

To avoid constructing the full foliation, one may simply project an initial condition $\textbf{x}_0$ orthogonally to an observed spectral submanifold $\mathcal {W}(E)$ to obtain $\textbf{p}_{0}$, but this may result in large errors if *E* and *F* are not orthogonal. In that case, $\mathcal {W}(E)$ may divert substantially from *E* (see [42, 43, 48] for a discussion of the limitations of this projection for general invariant manifolds).

A better solution is to project $\textbf{x}_{0}$ orthogonally to the slow spectral subspace *E* over which $\mathcal {W}(E)$ is a graph in an (often large) neighborhood of the fixed point. This approach assumes that *E* and *F* are nearly normal and $\mathcal {W}(E)$ is nearly flat. As the latter is typically the case for delay-embedded observables [2], orthogonal projection onto *E* has been the choice so far in data-driven SSM-based reduction via the SSMLearn algorithm [11]. This approach has produced highly accurate reduced-order models in a number of examples (see [3, 12, 13]). There are nevertheless examples in which the linear part of the dynamical system is significantly non-normal and hence *E* and *F* are not close to being orthogonal (see [6]).

**Fig. 2:**
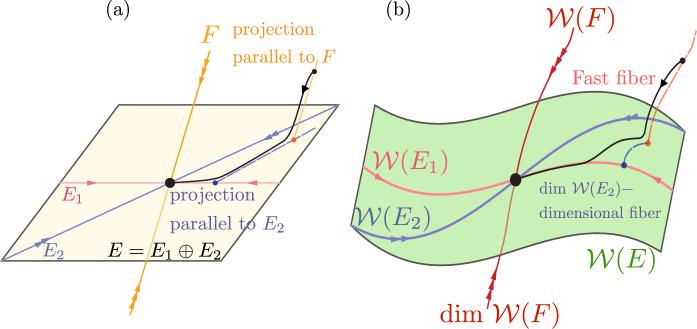
**a** The linearized phase space geometry governed by the slow spectral subspace $E=E_{1}\oplus E_{2}$ and the slow invariant foliation within *E*. **b**) Phase space geometry in the original coordinates

Near hyperbolic fixed points, the use of DDL eliminates the need to construct involved nonlinear spectral foliations. Indeed, let us assume that the slow spectral subspace *E* in Theorem 4 can be decomposed into a direct sum $E=E_{1}\oplus E_{2}$, where $E_{1}$ denotes the slowest spectral subspace with dim $E_{1}=d_{1}$ and $E_{2}$ denotes the second-slowest spectral subspace with dim $E_{2}=d_{2}$, as sketched in Fig. 2. Reducing the dynamics to the SSM $\mathcal {W}(E)$ is accurate for transient times given by the decay rate of $E_{2}.$ This initial reduction can be done simply by a normal projection onto *E*. Inside *E*, one can simply locate spectral foliations of the DDL-linearized systems explicitly and map them back to the original nonlinear system under the DDL transformation $\varvec{\kappa }\left( \varvec{\gamma }\right)$. The unique class $C^{a}$ foliation of a linear system within *E* is the family of stable fibers forming the affine space

$$\begin{aligned} \mathcal {F}_{\textbf{p}}=\textbf{p}+E_{2}, \end{aligned}$$

where $\textbf{p}\in E_{1}$. The trajectories started inside $\mathcal {F}_{\textbf{p}}$ all synchronize with $\textbf{p}\in E_{1}$. The linear projection $\textbf{P}_{E_{2}}$ onto $E_{1}$ along directions parallel to $E_{2}$, when applied to an initial condition $\textbf{y}\in \mathcal {F}_{\textbf{p}}$, returns the base point

51 $$\begin{aligned} \textbf{P}_{E_{2}}\textbf{y}=\textbf{p}. \end{aligned}$$

In the nonlinear system (1), the leaves of the smooth foliation within $\mathcal {W}(E)$

$$\begin{aligned} \mathcal {F}_{\varvec{\kappa }\left( \textbf{p}\right) }^{0}=\varvec{\kappa }\left( \mathcal {F}_{\textbf{p}}\right) \subset \mathcal {W}(E), \end{aligned}$$

where $\varvec{\kappa }\left( \textbf{p}\right) \in \mathcal {W}(E_{1})$ is the image of $\textbf{p}$ under the mapping $\varvec{\kappa }$ defined in (37). The SSM $\mathcal {W}(E)$ can then be parametrized via the foliation

$$\begin{aligned} \mathcal {W}(E)=\bigcup _{\textbf{q}\in \mathcal {W}(E_{1})}\mathcal {F}_{\textbf{q}}^{0}. \end{aligned}$$

#### Using DDL to predict nonlinear forced response from unforced data

We now discuss how DDL performed near the fixed point of an autonomous dynamical system can be used to predict nonlinear forced response under additional weak periodic forcing in the domain of DDL. The addition of such small forcing is frequent in structural vibration problems in which the unforced structure (e.g., a beam or disk) is rigid enough to react with small displacements under practically relevant excitation levels (see, e.g., [12, 13] for specific examples).

We append system (11) with a small, time-periodic forcing term $\varepsilon \textbf{F}(\textbf{x},t)$ to obtain the system

52 $$\begin{aligned} \dot{\textbf{x}}= & {} \textbf{A}\textbf{x}+\tilde{\textbf{f}}(\textbf{x})+\varepsilon \textbf{F}(\textbf{x},t),\qquad \textbf{x}\in {\mathbb {R}}^{n},\qquad \nonumber \\ \textbf{A}= & {} D\textbf{f}(0),\qquad \tilde{\textbf{f}}(\textbf{x})=\mathcal {O}\left( \left| \textbf{x}\right| ^{2}\right) ,\qquad 0\le \epsilon \ll 1, \end{aligned}$$

with $\textbf{F}(\textbf{x},t)=\textbf{F}(\textbf{x},t+T)$ for some period $T>0$. If the conditions of Theorem 4 hold for the system (52) for $\varepsilon =0$, then, for $\epsilon >0$ small enough, exists a unique *d*-dimensional, *T*-periodic, attracting spectral submanifold $\mathcal {W}_{\varepsilon }(E,t)\in C^{r}$ of a locally unique attracting *T*-periodic orbit $\textbf{x}_{\epsilon }(t)$ perturbing from $\textbf{x}=\textbf{0}$ (see, e.g., [10, 22]). The manifold $\mathcal {W}_{\varepsilon }(E,t)$ is $O(\varepsilon )$ $C^{1}$-close to $\mathcal {W}_{0}(E,t)\equiv \mathcal {W}(E)$ and hence its reduced dynamics can be parametrized using the reduced observable vector $\varvec{\varphi }=\varvec{\phi }\vert _{\mathcal {W}(E)}$ in the form

53 $$\begin{aligned}&\dot{\varvec{\varphi }} =\textbf{B}\varvec{\varphi }+\textbf{q}\left( \varvec{\varphi }\right) +\varepsilon \hat{\textbf{F}}(\varvec{\varphi },t),\qquad \nonumber \\&\textbf{B}=D\varvec{\phi }(\textbf{0})\textbf{T}_{E}\varvec{\Lambda }_{E}\left( D\varvec{\phi }(\textbf{0})\textbf{T}_{E}\right) ^{-1},\nonumber \\ {}&\textbf{q}\left( \varvec{\varphi }\right) =\mathcal {O}\left( \left| \varvec{\varphi }\right| ^{2}\right) ,\nonumber \\&\hat{\textbf{F}}(\varvec{\varphi },t)=\left( D\varvec{\phi }(\textbf{0})\textbf{T}_{E}\right) ^{-1}\left( \textbf{I}+D\textbf{h}\left( \varvec{\varphi },\textbf{0}\right) \right) ^{-1}\textbf{F}(\textbf{0},t)\nonumber \\&\quad +\mathcal {O}\left( \varepsilon \left| \varvec{\varphi }\right| ^{2}\right) , \end{aligned}$$

where we have relegated the details of this calculation to Appendix E.

Then the unique, $C^{r}$ change of coordinates,

54 $$\begin{aligned} \varvec{\varphi }=\varvec{\kappa }\left( \varvec{\gamma }\right) =\varvec{\gamma }+\varvec{\ell }(\varvec{\gamma }), \end{aligned}$$

guaranteed by statement (iii) of Theorem 4 transforms the reduced dynamics (53) to its final form

55 $$\begin{aligned} \dot{\varvec{\gamma }}=\textbf{B}\varvec{\gamma }+\varepsilon (\textbf{I}+D\varvec{\ell }(\varvec{\gamma }))^{-1}\hat{\textbf{F}}(\textbf{0},t). \end{aligned}$$

The transformation is valid on trajectories of (52) as long as they remain in the domain of definition of the coordinate change (54).

Note that Eq. (55) is a weakly perturbed, time-periodic nonlinear system. The matrix $\textbf{B}$ and the nonlinear terms $\varvec{\ell }(\varvec{\gamma })$ can be determined using data from the unforced ($\varepsilon =0$) system. As a result, nonlinear time-periodic forced response can be predicted *solely from unforced data* by applying numerical continuation to system (55) for $\varepsilon >0$. This is not expected to be as accurate as SSM-based forced response prediction (see, e.g., [2, 3, 12, 13]), but nevertheless offers a way to make predictions for non-linearizable forced response based solely on DDL performed on unforced data. These predictions are valid for forced trajectories that stay in the domain of convergence of DDL carried out on the unforced system. We will illustrate such predictions using actual experimental data from fluid sloshing in Sect. 5.4.

Setting $\varvec{\ell }(\varvec{\gamma })=\textbf{0}$ in formula (55) enables us to carry out a forced-response prediction based on DMD as well. Such a prediction will be fundamentally linear with respect to the forcing and can only be reasonably accurate for very small forcing amplitudes, as we will indeed see in examples. There is no systematic way to model the addition of non-autonomous forcing in the EDMD procedure, and hence EDMD will not be included in our forced response prediction comparisons.

We also note, that one might be tempted to solve an approximate version of (55) by assuming

56 $$\begin{aligned} \varepsilon (\textbf{I}+D\varvec{\ell }(\varvec{\gamma }))^{-1}\approx \varepsilon \textbf{I}. \end{aligned}$$

This assumption simplifies the computation of the forced response of the nonlinear system (55) to those of a simple linear system. Although the forced response computed using this approximate DDL method turns out to be more accurate than DMD on our example, we do not recommend this approach. This is because neglecting the nonlinear effects of the coordinate change in (55) is, in general, inconsistent with $\varvec{\ell }(\varvec{\gamma })\ne \textbf{0}$. We give more detail on this approximation in Appendix F of the Supplementary Information.

## Examples

In this section, we compare the DMD, EDMD and DDL algorithms on specific examples. When applicable, we also compute the exact analytic linearization of the dynamical system near its fixed point as a benchmark. On a slow SSM $\mathcal {W}\left( E\right)$, an observer trajectory $\varvec{\varphi }(t)$, starting from a select initial condition $\varvec{\varphi }(0)$, will be tracked as the image of the linearized reduced observer trajectory $\varvec{\gamma }(t)$ under the linearizing transformation (54):

57 $$\begin{aligned} \varvec{\varphi }(t)= & {} \varvec{\kappa }\left( e^{\textbf{B}t}\varvec{\gamma }(0)\right) =e^{\textbf{B}t}\varvec{\gamma }(0)+\varvec{\ell }\left( e^{\textbf{B}t}\varvec{\gamma }(0)\right) ,\qquad \nonumber \\ \varvec{\gamma }(0)= & {} \varvec{\kappa }^{-1}\left( \varvec{\varphi }(0)\right) . \end{aligned}$$

When model reduction has also taken place, i.e., when the observable vector $\varvec{\varphi }$ is not defined on the full phase space, we will nevertheless provide a prediction in the full phase space via the parametrization of the slow SSM.

By Theorem 1, DMD can be interpreted as setting $\varvec{\ell }(\varvec{\gamma })\equiv \varvec{0}$ in (57) and finding the linear operator $\textbf{B}$ as a best fit from the available data. In contrast, DDL finds the linear operator $\textbf{B}$, the transformation $\varvec{\varphi }=\varvec{\gamma }+\varvec{\ell }(\varvec{\gamma })$, and its inverse simultaneously. As we explained in Sect. 4.2, EDMD cannot quite be interpreted in terms of the linearizing transformation (57) as it is an attempt to immerse the dynamics into a higher dimensional space. For our EDMD tests, we will use monomials of the observable vector $\varvec{\varphi }$.

### 1D nonlinear system with two isolated fixed points

Consider the one-dimensional ODE obtained as the radial component of the Stuart–Landau equation, i.e.,

$$\begin{aligned} \dot{r}=\mu r-r^{3}, \end{aligned}$$

which can be rescaled to

58 $$\begin{aligned} \dot{R}=R-R^{3}. \end{aligned}$$

For $R\ge 0$, the system has a repelling fixed point at $R=0$ and an attracting one at $R=1$. Page and Kerswell [44] show that local expansion of observables in terms of the Koopman eigenfunctions computed near each fixed point are possible, but the expansions at the two fixed points are not compatible with each other and both diverge at $R=\sqrt{2}/2\approx 0.7071$. This is a consequence of the more general result that the Koopman eigenfunctions themselves inevitably blow up near basin boundaries (see our Proposition 1 in Appendix A of the Supplementary Information). Both DMD and EDMD can nevertheless be computed from data, even for a trajectory crossing the turning point at $R=\sqrt{2}/2$, but the resulting models cannot have any connection to the Koopman operator.

**Fig. 3:**
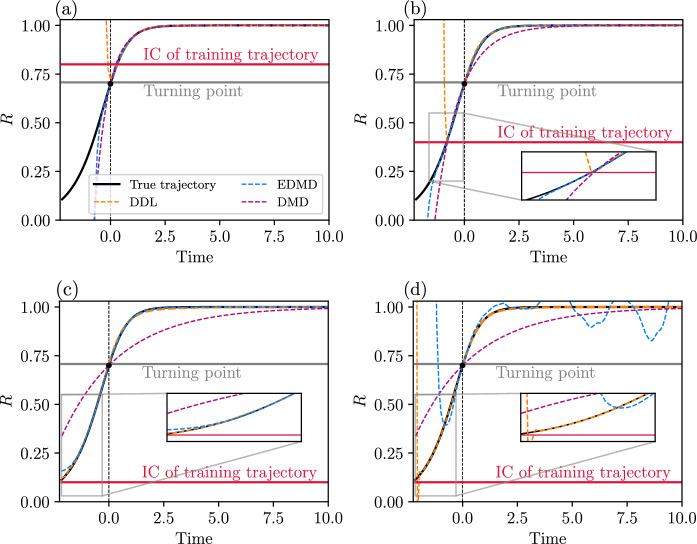
Predictions of DMD, EDMD and DDL on trajectories of (58). **a** Training trajectory starts inside the domain of convergence of the linearization, i.e. $R(0)=0.8$ (see [44]). For both DDL and EDMD the order of the monomials used is $k=5$. **b** Same for a different training trajectory with $R(0)=0.4$ and $k=5$ **c** same for $R(0)=0.1$ and $k=10$ **d** same for $R(0)=0.1$ and $k=18$

In each comparison performed on system (58), we generate a single trajectory in the domain of attraction of the $R=1$ fixed point and use it as training data for DMD, EDMD and DDL. In each subplot of Fig. 3, the single training trajectory starts from the intersection of the red horizontal line “IC of training trajectory” with the $t=0$ dashed line. We then also generate a new test trajectory (black) with its initial condition denoted with a black dot over the line $t=0$. We place this initial condition slightly outside the domain of linearization for system (58) (under the grey line labeled “Turning point”). We use DMD, order-$k=5$ EDMD, and DDL trained on a single training trajectory to make predictions for the black testing trajectory (not used in the training).

Figure 3a shows DDL to be the most accurate of the three methods when applied to forward-time ($t\ge 0$) segments of the test trajectory. If we try to predict the backward-time ($t<0$) segment of the same trajectory as it leaves the training domain, DDL diverges immediately upwards, whereas DMD and EDMD diverge more gradually downwards. As we increase the training domain in Fig 3b, DDL continues to be the most accurate in both forward and backward time until it reaches the domain of its training range in backward time. At that point, it diverges quickly upwards, while DMD and EDMD diverge more slowly downwards.

Importantly, increasing the approximation order for DDL first to $k=10$ then to $k=18$ (see Fig. 3c, d), makes DDL predictions more and more accurate in backward time inside the training domain. At the same time, the same increase in order makes EDMD less and less accurate inside the same domain. This is not surprising for EDMD because it seeks to approximate the dynamics within a Koopman-invariant subspaces for increasing *k*, and Koopman mode expansions blow up at the “Turning point line”, as shown both analytically and numerically by Page and Kerswell [44]. Interestingly, however, EDMD becomes less accurate even within the domain of linearization under increasing *k*. This is clearly visible in Fig. 3d which shows spurious, growing oscillations in the EDMD predictions close to the $R=1$ fixed point.

In summary, of the three methods tested, DDL makes the most accurate predictions in forward time. This remains true in backward time as longs as the trajectory remains in the training range used for the three methods, even if this range is larger than the theoretical domain of linearization. Inside the training range, an increase of the order *k* of the monomials used increases the accuracy of DDL but introduces growing errors in EDMD.

### 3D linear system studied via nonlinear observables

Wu et al. [60] studied the ability of DMD to recover a 3D linear system based on the time history of three nonlinear observables evaluated on the trajectories of the system. To define the linear system, they use a block-diagonal matrix $\varvec{\Lambda }$ and a basis transformation matrix $\textbf{R}$ of the form

59 $$\begin{aligned} \varvec{\Lambda }= & {} \left( \begin{array}{ccc} a &{} -b &{} 0\\ b &{} a &{} 0\\ 0 &{} 0 &{} c \end{array}\right) ,\quad a,b,c\in \mathbb {R},\qquad \nonumber \\ \textbf{R}= & {} \left( \begin{array}{ccc} 1 &{} 0 &{} \sin \theta _{1}\cos \theta _{2}\\ 0 &{} 1 &{} \sin \theta _{1}\sin \theta _{2}\\ 0 &{} 0 &{} \cos \theta _{2} \end{array}\right) , \end{aligned}$$

to define the linear discrete dynamical system

60 $$\begin{aligned} \textbf{x}(n+1)=\left( \textbf{R}\varvec{\Lambda }\textbf{R}^{-1}\right) \textbf{x}(n). \end{aligned}$$

The linear change of coordinates $\textbf{R}$ rotates the real eigenspace of $\varvec{\Lambda }$ corresponding to the eigenvalue *c* and hence introduces non-normality in system (60). This system is then assumed to be observed via a 3D nonlinear observable vector

61 $$\begin{aligned} \textbf{y}(\textbf{x})=\left( \begin{array}{c} x_{1}+0.1\left( x_{1}^{2}+x_{2}x_{3}\right) \\ x_{2}+0.1\left( x_{2}^{2}+x_{1}x_{3}\right) \\ x_{3}+0.1\left( x_{3}^{2}+x_{1}x_{2}\right) \end{array}\right) . \end{aligned}$$

Ideally, DMD should closely approximate the linear dynamics of system (60) because the observable function defined in Eq. (61) is close to the identity and has only weak nonlinearities. Wu et al. [60] find, however, that this system poses a challenge for DMD, which produced inaccurate predictions for the spectrum of $\textbf{R}\varvec{\Lambda }\textbf{R}^{-1}$.

**Fig. 4:**
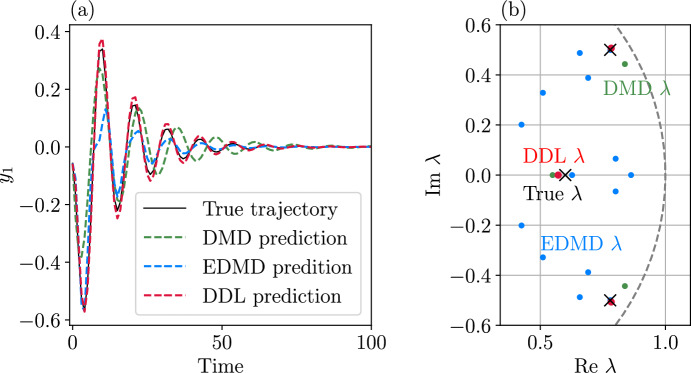
Predictions by DMD, EDMD, and DDL on the discrete dynamical system (59) and (61). **a** Predicted and true $y_{1}-$components of a test trajectory. **b** Spectra identified by DMD, EDMD, and DDL superimposed on the true spectrum (marked by crosses). The dashed line represents the unit circle. (Color figure online)

Following one of the parameter settings of Wu et al. [60], we set $a=0.45\sqrt{3}$, $b=0.5$, $c=0.6$, $\theta _{1}=1.5$, and $\theta _{2}=0$. We initialize three training trajectories with $\left\| \textbf{x}(0)\right\| <1,$ each containing 100 iterations of system (60). We then compute the predictions of a $5^{th}$ order DDL model and compare to those of DMD and EDMD on a separate test trajectory not used in training these three methods. The predictions and the spectrum obtained from the three methods are shown in Fig. 4.

The predictions of DMD and EDMD can only be considered accurate for very low amplitude oscillations, while DDL returns accurate predictions throughout the whole trajectory. This example consists of linear dynamics and monomial observables of the state, and hence should be an ideal test case for EDMD. Yet, EDMD is inaccurate in identifying the spectrum of system (60). Indeed, as seen in Fig. 4b, a number of spurious eigenvalues arise from EDMD, both real and complex. DMD performs clearly better but it is still markedly less accurate than DDL. These inaccuracies in the predictions of EDMD and DMD spectra are also reflected by considerable errors in their predictions for trajectories, as seen in Fig. 4a. In contrast, DDL produces the most accurate prediction for the test trajectory.

**Fig. 5:**
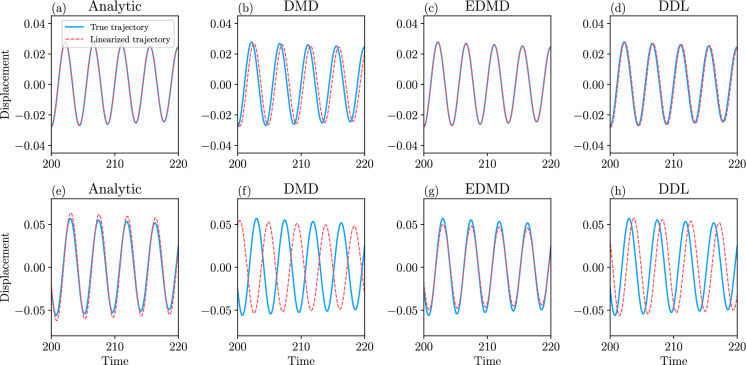
Comparison of the time evolution of the linearized trajectories (red) and the full trajectories of the nonlinear system (63) (blue), (63). **a**–**d** Analytic linearization, DMD, EDMD and DDL models trained and evaluated on trajectories inside the domain of convergence. **e**–**h** Same as (**a**)–(**d**) but outside the domain of convergence of the analytic linearization. (Color figure online)

### Damped and periodically forced Duffing equation

We consider the damped and forced Duffing equation

62 $$\begin{aligned} \dot{x}&=y,\nonumber \\ \dot{y}&=x-x^{3}-dy+\varepsilon \cos \Omega t, \end{aligned}$$

with damping coefficient $d=0.0141$, forcing frequency $\Omega$ and forcing amplitude $\varepsilon$. We perform a change of coordinates $\left( x,y\right) \mapsto \varvec{\varphi }=\left( \varphi _{1},\varphi _{2}\right)$ that moves the stable focus at $(x,y)=(1,0)$ to the origin and makes the linear part block-diagonal. The resulting system is of the form

63 $$\begin{aligned} \dot{\varvec{\varphi }}=\textbf{A}\varvec{\varphi }+\textbf{f}\left( \varvec{\varphi }\right) +\varepsilon \hat{\textbf{F}}(t),\qquad \textbf{f}\left( \varvec{\varphi }\right) =\mathcal {O}\left( \left| \varvec{\varphi }\right| ^{2}\right) , \end{aligned}$$

where

64 $$\begin{aligned} \textbf{A}=\begin{pmatrix}-\alpha &{} -\omega \\ \omega &{} -\alpha \end{pmatrix},\qquad \omega =1.4142,\quad \alpha =0.00707,\nonumber \\ \end{aligned}$$

and $\hat{\textbf{F}}(t)$ is the transformed image of the physical forcing vector in (62). We first consider the unforced system with $\varepsilon =0$. In this case, the 2D slow SSM of the fixed point coincides with the phase space $\mathbb {R}^{2}$ and hence no further model reduction is possible. However, since the non-resonance conditions (34) hold for the linear part (64), the system is analytically linearizable near the origin. The linearizing transformation and its inverse can both be computed from Eq. (63), as outlined in Eq. (41). For reference, we carry out this linearization analytically up to order $k=9$. The Taylor series of the linearization is estimated to converge for $\left| \varvec{\varphi }\right| <R_{crit}\approx 0.15$. The details of the calculation can be found in the repository [28].

We now compare the analytic linearization results it to DMD, EDMD and DDL, with all three trained on the same three trajectories, launched both inside and outside the domain of convergence of the analytic linearization. The polynomial order of approximation is $k=5$ for both the EDMD and the DDL algorithms. The performance of the various methods is compared in Fig. 5. Close to the fixed point, in the domain of convergence of the analytic linearization, all three methods perform well. Moving away from the fixed point, the analytic linearization is no longer possible. Both DMD and EDMD perform worse, while DDL continues to accurately linearize the system even outside the domain of convergence of the analytic linearization.

**Fig. 6:**
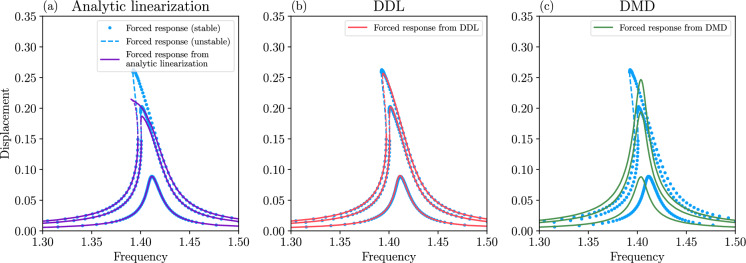
Periodic response of the Duffing oscillator under the forcing (63). The three distinctly colored forced response curves correspond to $\varepsilon =0.001,0.002,0.0028$. **a** Actual nonlinear forced response from numerical continuation (blue) and prediction for it from analytic linearization (purple) **b** forced response predictions from DDL (red) **c** forced response predictions from DMD (green). The training data for panels **b** and **c** is the same unforced trajectory data set as the one used in Fig. 5. (Color figure online)

Using formula (55) and our DDL-based model, we can also predict the response of system (63) for the forcing term of the form

65 $$\begin{aligned} \varepsilon \hat{\textbf{F}}(t)=\varepsilon \left( \begin{array}{c} -0.006\\ 1.225 \end{array}\right) \cos \Omega t, \end{aligned}$$

without using any data from the forced system. As the forced DDL model (55) is nonlinear, it can capture non-linearizable phenomena such as coexisting of stable and unstable periodic orbits arising under the forcing. We can also make a forced response prediction from DMD simply by setting $D\varvec{\ell }(\varvec{\gamma })=\varvec{0}$ in Eq. (55). As an inhomogeneous linear system of ODEs, however, this forced DMD model cannot predict coexisting stable and unstable periodic orbits.

In Fig. 6, we compare the forced predictions of the analytic linearization, DMD, and DDL to those computed from the nonlinear system directly via the continuation software COCO of Dankowicz and Schilder [14]. Since the forced and linearized systems are also nonlinear, we use the same continuation software to determine the stable and unstable branches of periodic orbits.

As expected, the analytic linearization is accurate while the forced response is inside the domain of convergence but deteriorates quickly for larger amplitudes. DMD gives good predictions for the peaks of the forced response diagrams, but cannot account for any of the nonlinear softening behavior, i.e., the overhangs in the curves that signal multiple coexisting periodic responses at the same forcing frequency. In contrast, while the DDL model of order $k=5$ starts becoming inaccurate for peak prediction at larger amplitudes outside the domain of analytic linearization, it continues to capture accurately the overhangs arising from non-linearizable forced response away from the peaks. Notably, DDL even identifies the unstable branches (in dashed lines) of the periodic response accurately. For completeness, we also show results of approximate DDL, by assuming (56) in Appendix F.

**Fig. 7:**
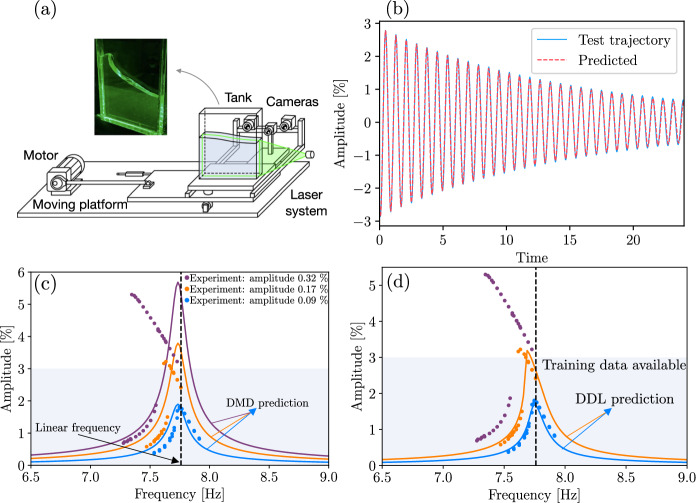
**a** Schematic representation of the experimental setup (adopted from [12]). **b** Prediction of the decay of a test trajectory with order-$k=5$ DDL. **c** Prediction of the forced response from DMD. **d** Prediction of the forced response from DDL. Light shading indicates the domain, in which training data for DMD and DDL was available

### Water sloshing experiment in a tank

In this section, we analyze experimental data generated by Bäuerlein and Avila [5] for forced and unforced fluid sloshing in a tank. Previous studies of this data set used nonlinear SSM-reduction to predict forced response [2, 3, 12]. Here we will use DMD and DDL to extract and compare linear reduced-order models from unforced trajectory data, then use them to predict and verify forced response curves obtained from forced trajectory data. Neither DMD nor DDL is expected to outperform the fully nonlinear approach of SSM reduction, so we will only compare them against each other.

The tank in the experiments is mounted on a platform that is displaced sinusoidally in time with various forcing amplitudes and frequencies (Fig. 7a). To train DMD and DDL, we use unforced sloshing data obtained by freezing the movement of the tank near a resonance and recording the ensuing decaying oscillations of the water surface with a camera under they die out. The resulting videos serve as input data to our analysis. Specifically, the horizontal position of the center of mass of the fluid is extracted from each video frame tracked and used as the single scalar observable.

During such a resonance decay experiment, the system approaches its stable unforced equilibrium via oscillations that are dominated by a single mode. In terms of the phase space geometry, this means an approach to a stable fixed point along its 2D slow SSM $\mathcal {W}\left( E\right)$ tangent to the slowest 2D real eigenspace *E* . As we only have a single observable from the videos, we use delay embedding to generate a larger observable space that can accommodate the 2D manifold $\mathcal {W}\left( E\right)$. As discussed by Cenedese et al. [12], we need an at least 5D observable space for this purpose by the Takens embedding theorem. In this space, $\mathcal {W}\left( E\right)$ turns out to be nearly flat for short delays (see [2]), which allows us to use a linear approximation for its parametrization. The reduced coordinates on $\mathcal {W}\left( E\right) \approx E$ can then be identified via a singular value decomposition of the data after one removes initial transients from the experimental data. The end of the transients can be identified as a point beyond which a frequency analysis of the data shows only one dominant frequency, the imaginary part of the eigenvalue corresponding to *E*.

All this analysis has been carried out using the publicly available SSMLearn package Cenedese et al. [11]. With $\mathcal {W}\left( E\right)$ identified, we use the DDL method with order $k=5$ to find the linearizing transformation and the linearized dynamics on $\mathcal {W}\left( E\right)$. In Fig 7b we show the prediction of the model on a decaying trajectory reserved for testing. The displacements are reported as percentage values, with respect to the depth of the tank. In Fig. 7c and d, we show predictions from DMD and DDL models for the forced response, compared with the experimentally observed response. Since the exact forcing function is unknown, we follow the calibration procedure outlined by Cenedese et al. [12] to find an equivalent forcing amplitude in the reduced-order model.

We present data for three forcing amplitudes. The DDL predictions are accurate up to $0.17\%$ amplitude, even capturing the softening trend. The largest-amplitude forcing resulted in response significantly outside the range of the training data; in this range, we were unable to find the converged forced response from DDL. We also show the corresponding DMD-predictions in Fig. 7c. Although the linear response can formally be evaluated for any forcing amplitude, DMD shows no trace of the softening trend, and is even inaccurate for low forcing amplitudes.

**Fig. 8:**
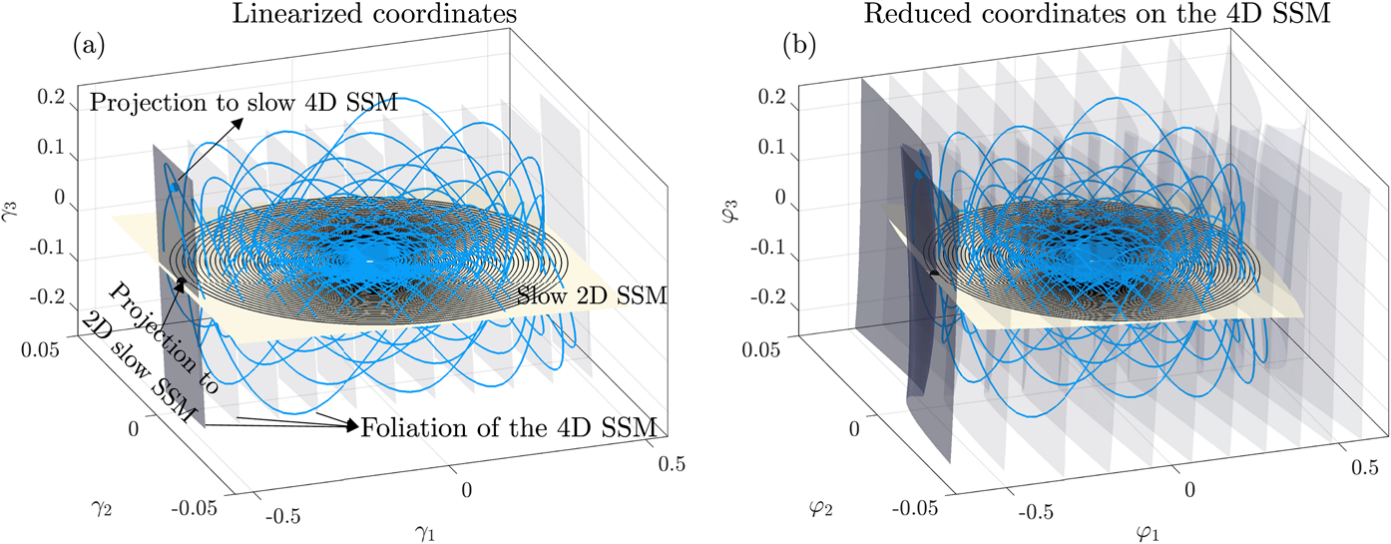
Reduced coordinates of the 4D SSM $\mathcal {W}(E)$ for the oscillator chain. The slow 2D SSM $\mathcal {W}(E_{1})$, a typical trajectory and its projection to the slow SSM along the fibers $\mathcal {F}_{\textbf{q}_{0}^{2D}}^{0}$ are also shown. Panel **a** shows the linearized coordinates computed from DDL, and **b** shows their image under the inverse of the linearizing transformation. The order of approximation used in DDL is 3

### Model reduction and foliation in a nonlinear oscillator chain

As a final example, we consider the dynamics of a chain of nonlinear oscillators, which has been analyzed in the SSMLearn package [11]. Denoting the positions of the oscillators as $q_{i}$ for $i=1,...,5$, we assume that the springs and dampers are linear, except for the first oscillator. The non-dimensionalized equations of motion can be written as

66 $$\begin{aligned} \textbf{M}\ddot{\textbf{q}}+\textbf{C}\dot{\textbf{q}}+\textbf{K}\textbf{q}+\textbf{f}(\textbf{q},\dot{\textbf{q}})=\textbf{0}, \end{aligned}$$

where $\textbf{M}=\textbf{I}$; the springs have the same linear stiffness $k=1$ which is encoded in $\textbf{K}$ via nearest-neighbor coupling. The damping is assumed to be proportional, i.e., we specifically set $\textbf{C}=0.002\textbf{M}+0.005\textbf{K}$.

Three numerically generated training trajectories show decay to the $$\textbf{q}=\textbf{0}$$ fixed point, as expected from the damped nature of the linear part of the system. In this example, we also seek to capture some of the transients, which motivates us to select the slow SSM $$\mathcal {W}(E)$$ to be 4D, tangent to the spectral subspace $$E=E_{1}\oplus E_{2}$$ spanned by the the slowest mode ($$E_{1})$$ and the second slowest mode ($$E_{2}$$). As the mode corresponding to $$E_{2}$$ does disappear over time from the decaying signal, there is no resonance between the eigenvalues and hence Theorem 4 is applicable. As already noted, numerical data from a generic physical system described by Eq. (66) will be free from resonances. An exception is a $$1:1$$ resonance arising from a perfect symmetry, but this resonance is not excluded by Theorem 4 and has is amenable to DDL.

Within the 4D SSM $\mathcal {W}(E)$, we also demonstrate how to optimally reduce the dynamics to its 2D slowest SSM $\mathcal {W}(E_{1})$. As explained in Sect. 4.3.3, to find the trajectory in $\mathcal {W}(E_{1})$ with which a given trajectory close to $\mathcal {W}(E)$ ultimately synchronizes, we need to project along a point $\textbf{q}_{0}$ of the full trajectory $\textbf{q}(t)$ first onto $\mathcal {W}(E)$ orthogonally to obtain a point $\textbf{q}_{0}^{4D}\in \mathcal {W}(E)$. We then need to identify the stable fiber $\mathcal {F}_{\textbf{q}_{0}^{2D}}$ in $\mathcal {W}(E)$ for which $\textbf{q}_{0}^{4D}\in \mathcal {F}_{\textbf{q}_{0}^{2D}}$ holds. Finally, one has to project along $\mathcal {F}_{\textbf{q}_{0}^{2D}}$ to locate its base point $\textbf{q}_{0}^{2D}\in \mathcal {W}(E_{1})$. The trajectory through $\textbf{q}_{0}^{2D}$ in $\mathcal {W}(E_{1})$ will then be the one with which the full trajectory $\textbf{q}(t)$ will synchronize faster than with any other trajectory. As noted in Sect. 4.3.3, computing the full nonlinear stable foliation

67 $$\begin{aligned} \mathcal {W}(E)=\bigcup _{\textbf{q}_{0}^{2D}\in \mathcal {W}(E_{1})}\mathcal {F}_{\textbf{q}_{0}^{2D}}^{0} \end{aligned}$$

of $\mathcal {W}(E)$ is simple in the linearized coordinates, in which it can be achieved via a linear projection along the faster eigenspace $E_{2}$.

We use a third-order polynomial approximation for $\mathcal {W}(E)$ based on the three training trajectories. The polynomials depend on the reduced coordinates we introduce along *E* using a singular value decomposition of the trajectory data. These reduced coordinates are shown in Fig. 8, where we show a representative training trajectory, the 2D slow SSM $E_{1}$, as well as the foliation (67) computed from DDL for this specific problem.

**Fig. 9:**
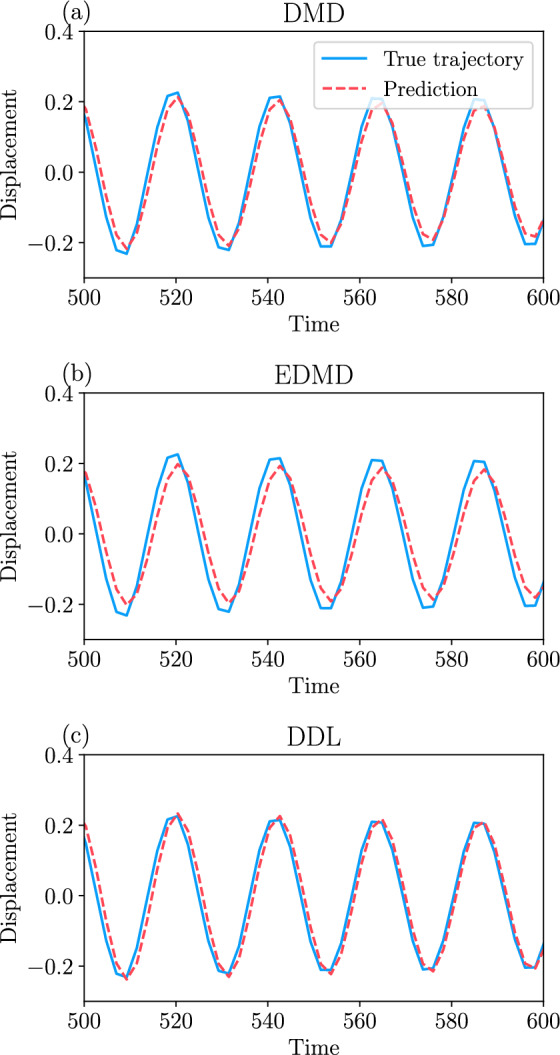
Predictions of **a** DMD **b** EDMD and **c** DDL models on a test trajectory of the oscillator chain. The order of approximation for DDL and EDMD is $k=3$

We also evaluate the DDL-based predictions on $\mathcal {W}(E)$ and $\mathcal {W}(E_{1})$ by comparing them to predictions from DMD and EDMD. Performing DMD and EDMD with the data first projected to *E* can be interpreted as finding the linear approximation to the dynamics in $\mathcal {W}(E)$. Similarly, performing DMD and EDMD with the data first projected to $E_{1}$ can be interpreted as finding the linear approximation to the dynamics in $\mathcal {W}(E_{1})$. These are to be contrasted with performing DDL that finds the linearized reduced dynamics within $\mathcal {W}(E)$, which in turn contains the linearized reduced dynamics within $\mathcal {W}(E_{1})$. Figure 9 shows that DMD and EDMD both perform similarly to DDL on $\mathcal {W}(E)$. However, the 2D DMD and EDMD results obtained for $\mathcal {W}(E_{1})$ are noticeably less accurate than the DDL results.

## Conclusions

We have given a new mathematical justification for the broadly used DMD procedure to eliminate the shortcomings of prior proposed justifications. Specifically, we have shown that under specific non-degeneracy conditions on the *n*-dimensional dynamical system, on $d\le n$ observable functions defined for that system, and on the actual data from these observables, DMD gives a leading-order approximation to the observable dynamics on an attracting *d*-dimensional spectral submanifold (SSM) of the system.

This result covers both discrete and continuous dynamical systems even for $n=\infty$. Our Theorem 1 only makes explicit non-degeneracy assumptions on the observables which will hold with probability one in practical applications. This is to be contrasted with prior approaches to DMD and its variants based on the Koopman operator, whose assumptions on the observables fail with probability one on generic observables.

Our approach also yields a systematic procedure that gradually refines the leading-order DMD approximation of the reduced observable dynamics on SSMs to higher orders. This procedure, which we call data-driven linearization (DDL), builds a nonlinear coordinate transformation under which the observable becomes linear on the attracting SSM. We have shown on several examples how DDL indeed outperforms DMD and extended DMD (EDMD), as expected. In addition to this performance increase, DDL also enables a prediction of truly nonlinear forced response from unforced data within its training range. Although we have only illustrated this for periodically forced water sloshing experiments in a tank, recent results on aperiodically time-dependent SSMs by Haller and Kaundinya [19] allow us to predict more general forced response using DDL trained on unforced observable data.

Despite all these advantages, DDL (as any linearization method) remains applicable only in parts of the phase space where the dynamics are linearizable. Yet SSMs continue to exist across basin boundaries and hence are able to carry characteristically nonlinear dynamics with multiple coexisting attractors. For such nonlinearizable dynamics, data-driven nonlinear SSM-reduction algorithms, such as SSMLearn and fastSSM, are preferable and have been showing high accuracy and predictive ability in a growing number of physical settings (see, e.g., [1, 3, 12, 13, 26, 27, 37]).

### Supplementary Information

Below is the link to the electronic supplementary material.

Supplementary file 1 (pdf 1978 KB)

## Data Availability

All data and codes used in this work are downloadable from the repository https://github.com/haller-group/DataDrivenLinearization.

## Footnotes

1 Assuming that each coordinate component $x_{j}$ of the full phase space vector $\textbf{x}$ of system (1) falls in a span of the same set of Koopman eigenfunctions $\left\{ \phi _{1}(\textbf{x}),\ldots ,\phi _{N}(\textbf{x})\right\}$, one defines the $j^{th}$ Koopman mode associated with $x_{j}$ as the vector $\textbf{v}_{j}=\left( v_{1j},\dots ,v_{Nj}\right) ^{\textrm{T}}$ for which $x_{j}=\sum _{i=1}^{N}v_{ij}\phi _{i}(x)$ (see, e.g., Williams et al. [58]).

2 More precisely, Fenichel’s foliation results become applicable after the wormhole construct in Proposition B1 of Eldering et al. [17] is applied to extend $\mathcal {W}(E)$ smoothly into a compact normally attracting invariant manifold without boundary. This is needed because Fenichel’s results only apply to compact normally attracting invariant manifolds with an empty or overflowing boundary, whereas the boundary of $\mathcal {W}(E)$ is originally inflowing.
