# Supplementary material for: Data-driven linearization of dynamical systems
Source: Nonlinear Dyn. 2024 Aug 15;112(21):18639–63. doi: 10.1007/s11071-024-10026-x (PMC11362512; doi:10.1007/s11071-024-10026-x)
Supplement: Supplementary file 1 — (pdf 1978 KB) [file 11071_2024_10026_MOESM1_ESM.pdf]

Supplementary Information for

Data-Driven Linearization of Dynamical Systems

George Haller[3] and Bálint Kaszás
Institute for Mechanical Systems
ETH Zürich
Leonhardstrasse 76, 8092 Zürich, Switzerland

A DMD and the Koopman operator

A.1 Observable dynamics and the Koopman operator

A classic approach, introduced first by Koopman [31] and revived recently by multiple authors (see, e.g., by Budišić et al. [8], Mezić [40], Kutz et al. [32] and the references cited therein), describes observable evolution via the Koopman operator $\mathcal{K}^t\colon \mathcal{G}^d \to \mathcal{G}^d$, defined on a Banach space $\mathcal{G}^d$ of d-dimensional observable functions as the pull-back operation on observables under the flow map $\mathbf{F}^t$ of system (1). Specifically,

$$\mathcal{K}^t\left[\boldsymbol{\phi}\right](\mathbf{x}_0) := \boldsymbol{\phi}\left(\mathbf{F}^t(\mathbf{x}_0)\right), \tag{68}$$

or, in more compact notation,

$$\mathcal{K}^t\left[\boldsymbol{\phi}\right] = \boldsymbol{\phi}\circ\mathbf{F}^t, \quad \boldsymbol{\phi}\in\mathcal{G}^d. \tag{69}$$

As $\mathcal{G}^d$ is a complete vector space, linear combinations of ℓ observables $\phi_1,\dots,\phi_\ell\in\mathcal{G}^d$ are also observables in $\mathcal{G}^d$ and hence $\mathcal{K}^t$ can be evaluated on them. Such an evaluation gives

$$\mathcal{K}^t\left(c_1\phi_1+\ldots+c_1\phi_\ell\right)(\mathbf{x}_0) = c_1\mathcal{K}^t\left[\phi_1\right](\mathbf{x}_0)+\ldots+c_\ell\mathcal{K}^t\left[\phi_\ell\right](\mathbf{x}_0) \tag{70}$$

This last equation shows that the mapping $\mathcal{K}^t$ is linear with respect to the choice of observables while the system's initial condition $\mathbf{x}_0$ is kept fixed. In contrast, DMD seeks to find an approximate mapping that is linear with respect to the choice of the system's initial conditions while the observable is kept fixed. Indeed, in applications of DMD, eq. (6) is posed for a fixed observable vector evaluated on various initial conditions. EDMD is also trained on system trajectories launched from different initial conditions, observed under the same general function of an initially fixed set of observables.

On a broader note, the linearity of the Koopman operator with respect to changes in the choice of an observable, as seen in eq. (70), does not imply linear dynamics for observations of the system under a fixed observable. For instance, let $\phi_1(\mathbf{x})$ denote the observation of the displacement, measured in meters, of the end point of a nonlinear beam whose current state in its phase space is denoted by $\mathbf{x}$. If, instead, we want to observe the position of the endpoint in millimeters, then we switch the observable $\phi_2(\mathbf{x}) = 10^3\phi_1(\mathbf{x})$, and hence for the observed initial state $\mathbf{x}_0$ of the beam, we have the change

$$\phi_2(\mathbf{x}_0) = c\phi_1(\mathbf{x}_0), \qquad c = 10^3,$$

in the initial observation. It is then no surprise that this change in the initial observable will transform to the same change in the current observations of the endpoint at time t, i.e.,

$$\mathcal{K}^t\left[\phi_2\right](\mathbf{x}_0) = c\mathcal{K}^t\left[\phi_1\right](\mathbf{x}_0).$$

This simple fact, however, just reflects that the final results at time t change by the same multiple as their initial conditions if we decide to change the physical units in our measurements. Clearly, this fact does not imply any linear dynamics for the displacement of the endpoint of the beam. Indeed, $\mathcal{K}^t\left[\phi_2\right](\mathbf{x}_0)$ may well be a chaotic signal.

[3]Corresponding author. Email: georgehaller@ethz.ch

Another example would be tracking the total population of a continent and denoting the current state of the population by $\mathbf{x}$. Let $\phi_1(\mathbf{x})$ denote specifically the observable returning the population of country A and $\phi_2(\mathbf{x})$ denote the observable returning the population of country B within the same continent. If we decide now to jointly observe the population of these two countries, then we are in effect passing to a third observable at at initial state $\mathbf{x}_0$ to obtain

$$\phi_3(\mathbf{x}_0) = \phi_2(b_0) + \phi_1(\mathbf{x}_0),$$

for the initial observations. To obtain the total population of countries A and B at the current state $\mathbf{x}$ of the total population, we can simply write

$$\boldsymbol{\mathcal{K}}^t\left[\phi_3\right](\mathbf{x}_0) = \boldsymbol{\mathcal{K}}^t\left[\phi_1\right](\mathbf{x}_0) + \boldsymbol{\mathcal{K}}^t\left[\phi_2\right](\mathbf{x}_0).$$

Again, this fact by itself does not say anything about the linearity or nonlinearity of the population growth in either country A or country B.

Finally, *the Koopman operator is as much nonlinear as it is linear with respect to the changes in the observables* as it commutes with any nonlinear single or multi-variable function of observables, as longs as that function is also an observable. Indeed, for any function

$$\mathbf{g}\colon \left(\mathcal{G}^d\right)^\ell \to \mathcal{G}^d,$$

the observable $\boldsymbol{\psi}(\mathbf{x}_0) = \mathbf{g}\left(\phi_1(\mathbf{x}_0), \ldots, \phi_\ell(\mathbf{x}_0)\right)$ satisfies

$$\boldsymbol{\mathcal{K}}^t\left[\mathbf{g}\left(\phi_1, \ldots, \phi_\ell\right)\right] = \mathbf{g}\left(\boldsymbol{\mathcal{K}}^t\left[\phi_1\right], \ldots, \boldsymbol{\mathcal{K}}^t\left[\phi_\ell\right]\right).$$

To illustrate this general property of the Koopman operator, let $\mathbf{x}$ denote the instantaneous velocity field of a 2D Navier-Stokes flow with density ρ and let $\phi_{ij}(\mathbf{x}) = |v_{ij}|$ be the specific velocity magnitude along the (i,j) location of a fixed spatial grid. If we now want to track the local kinetic energy of the flow at (i,j), we do not have to reformulate and re-solve the Navier–Stokes equation for $\phi_{\text{kin}}(\mathbf{x}) = \frac{1}{2}\rho\phi_{ij}^2(\mathbf{x}) = |x_{ij}|^2$. Rather, we can use our already available observations of $\phi_{ij}(\mathbf{x})$ of the velocity field starting from an initial velocity field $\mathbf{x}_0$ to obtain $\boldsymbol{\mathcal{K}}^t\left[\phi_{\text{kin}}\right](\mathbf{x}_0) = \frac{1}{2}\rho\left[\boldsymbol{\mathcal{K}}^t\left[\phi_{ij}\right](\mathbf{x}_0)\right]^2$. This, however, does not imply a statement that "the dynamics of the 2D Navier–Stokes equation are quadratic in the space of observables".

A.2 Differential equation for the dynamics of observables under the Koopman operator

We can directly verify that

$$\boldsymbol{\mathcal{K}}^{t_1+t_2}\boldsymbol{\phi} = \boldsymbol{\mathcal{K}}^{t_2}\boldsymbol{\mathcal{K}}^{t_1}\boldsymbol{\phi} = \boldsymbol{\mathcal{K}}^{t_1}\boldsymbol{\mathcal{K}}^{t_2}\boldsymbol{\phi}, \quad \left(\boldsymbol{\mathcal{K}}^t\right)^{-1}\boldsymbol{\phi} = \boldsymbol{\mathcal{K}}^{-t}\boldsymbol{\phi},$$

i.e., $\boldsymbol{\mathcal{K}}^t$ defines a flow map on the observable space $\mathcal{G}^d$, as long as system (1) generates a flow $\mathbf{F}^t$ on the phase space $\mathcal{P}$. To describe the dynamics generated by this flow map, let us select an arbitrary scalar observable $\boldsymbol{\phi} \in \mathcal{G}^d$ and differentiate the defining relation (68) with respect to t to obtain

$$\frac{d}{dt}\left\{\boldsymbol{\mathcal{K}}^t\left[\boldsymbol{\phi}\right](\boldsymbol{x}_0)\right\} = D\boldsymbol{\phi}\left(\mathbf{F}^t(x_0)\right)\mathbf{f}\left(\mathbf{F}^t(x_0)\right). \tag{71}$$

Since we have

$$D_{\mathbf{x}_0}\boldsymbol{\phi}\left(\mathbf{F}^t(\mathbf{x}_0)\right) = D\boldsymbol{\phi}\left(\mathbf{F}^t(\mathbf{x}_0)\right)D\mathbf{F}^t(\mathbf{x}_0),$$

and $\mathbf{F}^t$ is a diffeomorphism, we can rewrite (71) as

$$\frac{d}{dt}\left\{\boldsymbol{\mathcal{K}}^t\left[\boldsymbol{\phi}\right](\mathbf{x}_0)\right\} = D_{\mathbf{x}_0}\boldsymbol{\phi}\left(\mathbf{F}^t(\mathbf{x}_0)\right)\left[D\mathbf{F}^t(\mathbf{x}_0)\right]^{-1}\mathbf{f}\left(\mathbf{F}^t(\mathbf{x}_0)\right) = D_{\mathbf{x}_0}\left\{\boldsymbol{\mathcal{K}}^t\left[\boldsymbol{\phi}\right](\mathbf{x}_0)\right\}\left[D\mathbf{F}^t(\mathbf{x}_0)\right]^{-1}\mathbf{f}\left(\mathbf{F}^t(\mathbf{x}_0)\right). \tag{72}$$

Note, however, that

$$\left[D\mathbf{F}^t(\mathbf{x}_0)\right]^{-1}\mathbf{f}\left(\mathbf{F}^t(\mathbf{x}_0)\right)=\mathbf{f}(\mathbf{x}_0), \tag{73}$$

where we used the fact that $\mathbf{f}\left(\mathbf{F}^t(\mathbf{x}_0)\right)$ is a solution of the equation of variations $\dot{\boldsymbol{\xi}}=D\mathbf{F}^t(\mathbf{x}_0)\boldsymbol{\xi}$ and hence we have $\mathbf{f}\left(\mathbf{F}^t(\mathbf{x}_0)\right)=D\mathbf{F}^t(\mathbf{x}_0)\mathbf{f}\left(\mathbf{x}_0\right)$. Therefore, we obtain from eqs. (72) and (73) that

$$\frac{d}{dt}\left(\boldsymbol{\mathcal{K}}^t\boldsymbol{\phi}\right)=\boldsymbol{\mathcal{L}}\boldsymbol{\mathcal{K}}^t\boldsymbol{\phi} \tag{74}$$

with the Liouville operator $\boldsymbol{\mathcal{L}}:\mathcal{G}^d\mapsto\mathcal{G}^d$ defined as

$$\boldsymbol{\mathcal{L}}\colon\boldsymbol{\phi}\mapsto\boldsymbol{L}\boldsymbol{\phi}:=D\boldsymbol{\phi}\,\mathbf{f}.$$

A.3 An example: The phase space variable as observable

As an illustration of the solution structure of the functional differential equation (74), consider the simplest nontrivial case wherein the observable function $\boldsymbol{\phi}$ is just the identity map on $\mathcal{P}=\mathbb{R}^d$, i.e., $\hat{\boldsymbol{\phi}}=\mathbf{I}\colon\mathbb{R}^d\to\mathbb{R}^d$. In that case, we specifically have the expressions

$$\hat{\boldsymbol{\phi}}(\mathbf{x}_0)=\mathbf{x}_0,\qquad \boldsymbol{\mathcal{K}}^t\hat{\boldsymbol{\phi}}(\mathbf{x}_0)=\mathbf{F}^t(\mathbf{x}_0),\qquad \frac{d}{dt}\left\{\boldsymbol{\mathcal{K}}^t\hat{\boldsymbol{\phi}}\right\}=\dot{\mathbf{F}}^t(\mathbf{x}_0)=\dot{\mathbf{x}},\qquad D_{\mathbf{x}_0}\left\{\boldsymbol{\mathcal{K}}^t\hat{\boldsymbol{\phi}}(\mathbf{x}_0)\right\}=D\mathbf{F}^t(\mathbf{x}_0).$$

Substituting these formulas into the functional differential equation (74), we obtain

$$\dot{\mathbf{x}}=D\mathbf{F}^t(\mathbf{x}_0)\mathbf{f}(\mathbf{x}_0)=\mathbf{f}(\mathbf{x}). \tag{75}$$

Therefore, for the most commonly used observable in classic dynamical systems, the phase space variable $\mathbf{x}$, the linear functional differential equation (74) simplifies to the original nonlinear ODE ((1)).

This again underscores that the linearity of the Koopman operator does not imply linear dynamics for individual observations $\mathbf{x}(t;\mathbf{x}_0)$ of the evolution of the initial condition $\mathbf{x}_0$. Rather, it implies that for any real constant c, *the scaled initial observation* $\tilde{\boldsymbol{\phi}}\left(\mathbf{x}_0\right)=c\hat{\boldsymbol{\phi}}(\mathbf{x}_0)$ *of* $\mathbf{x}_0$ *will evolve into the identically scaled current observation* $\boldsymbol{\mathcal{K}}^t\tilde{\boldsymbol{\phi}}(\mathbf{x}_0)=c\boldsymbol{\mathcal{K}}^t\hat{\boldsymbol{\phi}}(\mathbf{x}_0)=c\mathbf{x}(t;\mathbf{x}_0)$ *of the trajectory at time* t, i.e., $\boldsymbol{\mathcal{K}}^t$ will return $\mathbf{x}(t;\mathbf{x}_0)$ scaled by the same constant c. Most importantly, we will still generally have

$$\mathbf{x}(t;c\mathbf{x}_0)\neq c\mathbf{x}(t;\mathbf{x}_0),$$

unless the dynamical system (75) is linear. Therefore, the dynamics of the fixed observable $\mathbf{x}(t;\mathbf{x}_0)$ is not linear in the usual sense, i.e., with respect to changes in initial conditions. Rather, it is linear with respect to changes in the observable in which the evolution of the same trajectory is observed.

Equation (75) also illustrates that passing from the autonomous nonlinear ODE (1) to the functional differential equation (74) gives as infinite-dimensional linear formulation that is at least as complicated to solve as the original finite-dimensional nonlinear ODE formulation.

A.4 Koopman eigenfunctions

There are, nevertheless, non-generic sets of observables restricted to which e.q. (74) becomes a constant-coefficient linear system of ODEs. Examples are observables falling in the span of eigenfunctions of the Koopman operator $\boldsymbol{\mathcal{K}}^t$, if and where such eigenfunctions exist.

Indeed, if for some constant $\lambda\in\mathbb{C}$, the eigenvalue problem

$$\boldsymbol{\mathcal{K}}^t\left[\boldsymbol{\phi}\right](\mathbf{x}_0)=e^{\lambda t}\boldsymbol{\phi}(\mathbf{x}_0),\quad \mathbf{x}_0\in\mathcal{D}, \tag{76}$$

has a solution $\boldsymbol{\phi}\colon\mathcal{D}\to\mathbb{C}^d$ over some domain $\mathcal{D}\subset\mathcal{P}$ of the phase space, then we obtain

$$\frac{d}{dt}\boldsymbol{\mathcal{K}}^t\left[\boldsymbol{\phi}\right]=\lambda\boldsymbol{\mathcal{K}}^t\left[\boldsymbol{\phi}\right].$$

Consequently, if $\boldsymbol{\phi}_1(\boldsymbol{x}_0),\ldots,\boldsymbol{\phi}_k(\boldsymbol{x}_0)\in\mathbb{C}^d$ are Koopman eigenfunctions with corresponding Koopman eigenvalues $\lambda_1,\ldots,\lambda_k\in\mathbb{C}$ and domains of definition $\mathcal{D}_1,\ldots,\mathcal{D}_k\subset\mathcal{P}$, then any observable $\boldsymbol{w}\in\oplus^d\left(\cap_{j=1}^k\mathcal{D}_j\right)$ of the form

$$\boldsymbol{w}(\mathbf{x}_0)=c_1\boldsymbol{\phi}_1(\mathbf{x}_0)+\ldots+c_k\boldsymbol{\phi}_k(\mathbf{x}_0) \tag{77}$$

satisfies

$$\boldsymbol{\mathcal{K}}^t\left[\boldsymbol{w}\right]=\sum_{j=1}^k c_j\boldsymbol{\mathcal{K}}^t\left[\boldsymbol{\phi}^j\right]=\sum_{j=1}^k c_je^{\lambda_jt}\boldsymbol{\phi}_j.$$

Therefore, the coordinate representation $\boldsymbol{W}(t)$ of $\boldsymbol{\mathcal{K}}^t\left[\boldsymbol{w}\right]$ in the basis $\boldsymbol{\phi}_1,\ldots,\boldsymbol{\phi}_j$, given by

$$\mathbf{W}(t)=\begin{pmatrix} c_1e^{\lambda_1t}\\ \vdots\\ c_ke^{\lambda_kt}\end{pmatrix},$$

satisfies the k-dimensional autonomous linear ODE

$$\frac{d}{dt}\mathbf{W}=\boldsymbol{\Lambda}\mathbf{W},\quad \boldsymbol{\Lambda}=\begin{pmatrix}\lambda_1 & 0 & 0\\ 0 & \ddots & 0\\ 0 & 0 & \lambda_k\end{pmatrix}.$$

More generally, if $\boldsymbol{\phi}_1,\ldots,\boldsymbol{\phi}_k$ are just linearly independent observables in the spectral subspace span $\{\boldsymbol{\phi}_1,\ldots,\boldsymbol{\phi}_k\}$, then a similar ODE holds for W in the basis $\boldsymbol{\phi}_1,\ldots,\boldsymbol{\phi}_k$. In that case, Λ is not a diagonal matrix but its eigenvalues are still $\lambda_1,\ldots,\lambda_k$.

It is often forgotten, however, that Koopman eigenfunctions satisfying the eigenvalue problem (76) generally only exist on a subset $\mathcal{D}$ of the phase space. This is made precise by the following simple observation:

Proposition 1. *At least one principal Koopman eigenfunction blows up (i.e., becomes unbounded) at the boundary of a domain of attraction or repulsion of a fixed point around which the underlying dynamical system admits a local C^1linearization.*

Proof. We only prove the statement for domains of attraction of fixed points of continuous dynamical systems. Domains of repulsion can be handled in the same fashion in backward time and the proof for discrete dynamical systems is similar. Let $\mathbf{x}_0$ be a point on the boundary $\partial\mathcal{B}$ of a domain of attraction $\mathcal{B}$ of a fixed point p of a continuous dynamical system. Then, arbitrarily close to $\mathbf{x}_0$, there are initial conditions $\hat{\mathbf{x}}_0$ in the domain of attraction with arbitrarily long times of flight to the ball $B_\rho(p)$ around p in which the dynamical system can be C^1linearized.

Lan and Mezic [35] extended local C^1 linearization near an attracting fixed point via the backward-time flow map to a global C^1 linearizing transformation $\mathbf{x}=\mathbf{h}(\mathbf{y})$ within $\mathcal{B}$ that maps the original ODE within $\mathcal{B}$ to $\dot{\mathbf{y}}=\mathbf{A}\mathbf{y}$ defined on $\mathbb{R}^n$. By the construction of this linearizing transformation, initial conditions $\hat{\mathbf{x}}_0$ whose times of flight to $B_\rho(p)$ is large will be mapped into initial conditions $\hat{\mathbf{y}}_0$ of the linearized system that are far from the origin $\mathbf{y}=\mathbf{0}$.

The unique principal Koopman eigenfunctions of the nonlinear system are known to be

$$\boldsymbol{\phi}_j(\mathbf{x})=\left\langle\mathbf{h}^{-1}(\mathbf{x}),\mathbf{v}_j\right\rangle,\quad j=1,\ldots,n, \tag{78}$$

with $\mathbf{v}_j$ denoting the left eigenvectors or $\mathbf{A}$. As we have just concluded that $\mathbf{y}=\mathbf{h}^{-1}(\mathbf{x})$ will admit arbitrarily large values arbitrarily close to any point $\mathbf{x}_0\in\partial\mathcal{B}$, it follows that there will be at least one j for which the principal Koopman eigenfunction $\boldsymbol{\phi}_j(\mathbf{x})$ blows up at $\mathbf{x}_0\in\partial\mathcal{B}$. □

We note that the statement of Proposition 1 can also be deduced from the more general results in Theorem 3 of Kvalheim and Arathoon [33].

As a consequence of Proposition 1, one cannot simply patch together Koopman eigenfunctions outside boundaries of domains of attraction and thereby provide a viable Koopman-linearization for the whole phase space, as is often suggested. Indeed, even if found somehow, Koopman eigenfunctions would become unmanageable for the purposes of observer expansions even before reaching basin boundaries, as shown explicitly by the examples of Page and Kerswell [44].

B Proof of Theorem 1 and technical remarks

Under assumption (A1) of hyperbolicity of the theorem and under assumption (A2) on the smoothness class C^2 of the dynamical system, a refinement of the Hartman–Grobman theorem (see Guckenheimer and Holmes [19]) by Hartman [23] guarantees the local existence of a near-identity, linearizing change of coordinates of the form[4]

$$\mathbf{x} = \mathbf{y} + \mathbf{h}(\mathbf{y}), \qquad \mathbf{y} \in U \subset \mathbb{R}^n, \qquad \mathbf{h}(\mathbf{y}), \mathbf{h}^{-1}(\mathbf{y}) = \mathcal{O}\left(|\mathbf{y}|^{1+\beta}\right), \tag{79}$$

for some $\beta \in (0,1)$, under which system (11) takes the exact linear form

$$\dot{\mathbf{y}} = \mathbf{A}\mathbf{y}. \tag{80}$$

As pointed out by Haller et al. [22] in the context of the more restrictive, C^∞ linearization theorem of Sternberg [53], the inverse of the linearizing transformation (79) maps the d-dimensional spectral subspace E of the linearized system (80) into a d-dimensional, normally attracting spectral submanifold (SSM),

$$\mathcal{W}(E) = \left(\mathbf{I} + \mathbf{h}\right)^{-1}(E), \tag{81}$$

for system (11). The internal dynamics of this SSM govern the longer-term behavior of all trajectories near the origin. Generally, there will be infinitely many such invariant manifolds, but they are all tangent to E at the origin. We note that, $\mathcal{W}(E)$, as defined in eq. (81), is only known to be C^1 under our current set of assumptions. Under further nonresonance assumption on the spectrum of $\mathbf{A}$, the SSM can be shown to be as smooth as the underlying dynamical system (see Cabré et al. [10], Haller and Ponsioen [21]).

Based on these results, we apply the linearizing transformation (79) followed by a linear change of coordinates to $(\boldsymbol{\xi}, \boldsymbol{\eta})$, where $\boldsymbol{\xi}$ are coordinates along the spectral subspace E and $\boldsymbol{\eta}$ are coordinates along the spectral subspace F. Specifically, let

$$\mathbf{x} = \mathbf{T}\mathbf{y} + \mathbf{h}(\mathbf{T}\mathbf{y}), \quad \mathbf{y} = (\boldsymbol{\xi}, \boldsymbol{\eta})^{\mathrm{T}} \in \mathbb{R}^d \times \mathbb{R}^{n-d}, \tag{82}$$

with the matrix $\mathbf{T}$ defined in formula (15).

Under the transformation (82), the nonlinear system (11)becomes

$$\begin{pmatrix} \dot{\boldsymbol{\xi}} \\ \dot{\boldsymbol{\eta}} \end{pmatrix} = \begin{pmatrix} \boldsymbol{\Lambda}_E & 0 \\ 0 & \boldsymbol{\Lambda}_F \end{pmatrix} \begin{pmatrix} \boldsymbol{\xi} \\ \boldsymbol{\eta} \end{pmatrix}, \qquad \boldsymbol{\Lambda}_E = \left(\mathbf{T}^{-1}\mathbf{A}\mathbf{T}\right)|_E, \qquad \boldsymbol{\Lambda}_F = \left(\mathbf{T}^{-1}\mathbf{A}\mathbf{T}\right)|_F. \tag{83}$$

In these coordinates, the smoothest invariant manifold tangent to E is

$$\mathcal{W}(E) = \left\{(\boldsymbol{\xi}, \boldsymbol{\eta}) \,:\, \boldsymbol{\eta} = 0\right\},$$

within which the dynamics restricted to $\mathcal{W}(E)$ are then given by

$$\dot{\boldsymbol{\xi}} = \boldsymbol{\Lambda}_E \boldsymbol{\xi}. \tag{84}$$

[4] Hartman [23] only states that $h \in C^1$ in his main theorem. He adds, however, that his proof of the theorem also shows that Dh is uniformly Hölder continuous in U with Hölder exponent $\beta \in (0,1)$, which implies eq. (79).

From eq. (83), we obtain the estimates

$$|\boldsymbol{\xi}(t;\boldsymbol{\xi}_0)| \leq |\boldsymbol{\xi}_0| e^{(\mathrm{Re}\lambda_1 + \epsilon_1)t},$$
$$|\boldsymbol{\eta}(t;\boldsymbol{\eta}_0)| \leq |\boldsymbol{\eta}_0| e^{(\mathrm{Re}\lambda_{d+1} + \epsilon_{d+1})t},$$

where $\epsilon_j \geq 0$ is an arbitrarily small constant that can be chosen zero if the algebraic multiplicity of λ_j is equal to its geometric multiplicity.

With the help of the $(\boldsymbol{\xi},\boldsymbol{\eta})$ coordinates and eq. (79), the class C^2 observable $\boldsymbol{\phi}$ be locally be written as

$$\begin{aligned}
\boldsymbol{\phi}(x) &= \boldsymbol{\phi}(\mathbf{Ty}+\mathbf{h}(\mathbf{Ty})) = D\boldsymbol{\phi}(\mathbf{0})\left[\mathbf{Ty}+\mathbf{h}(\mathbf{Ty})\right] + \mathcal{O}\left(|\mathbf{y}+\mathbf{h}(\mathbf{y})|^2\right) \\
&= D\boldsymbol{\phi}(\mathbf{0})\mathbf{T}\begin{pmatrix}\boldsymbol{\xi}\\ \boldsymbol{\eta}\end{pmatrix} + D\boldsymbol{\phi}(\mathbf{0})\mathbf{h}(\mathbf{Ty}) + \mathcal{O}\left(|\boldsymbol{\xi}|^2, |\boldsymbol{\xi}|\,|\boldsymbol{\eta}|, |\boldsymbol{\eta}|^2\right) \\
&= D\boldsymbol{\phi}(\mathbf{0})\mathbf{T}_E\boldsymbol{\xi} + \mathcal{O}(|\boldsymbol{\eta}|) + \mathcal{O}\left(|(\boldsymbol{\xi},\boldsymbol{\eta})|^{1+\beta}\right) + \mathcal{O}\left(|\boldsymbol{\xi}|^2, |\boldsymbol{\xi}|\,|\boldsymbol{\eta}|, |\boldsymbol{\eta}|^2\right) \\
&= D\boldsymbol{\phi}(\mathbf{0})\mathbf{T}_E\boldsymbol{\xi} + \mathcal{O}(|\boldsymbol{\eta}|) + \mathcal{O}\left(|(\boldsymbol{\xi},\boldsymbol{\eta})|^{1+\beta}\right)
\end{aligned} \tag{85}$$

for some $\beta \in (0,1)$.

We also express the data matrix $\boldsymbol{\Phi}$ defined in eq. (5) in terms of data matrices with respect to the coordinates $\mathbf{y} = (\boldsymbol{\xi},\boldsymbol{\eta})$ by letting

$$\boldsymbol{\Phi} = \boldsymbol{\phi}(\mathbf{TY}-\mathbf{h}(\mathbf{TY})), \qquad \mathbf{Y} = \begin{pmatrix}\boldsymbol{\Xi}\\ \boldsymbol{H}\end{pmatrix}, \tag{86}$$

where the functions $\boldsymbol{\phi}$ and $\boldsymbol{h}$ are applied to the matrices involved column by column. From eqs. (85) and (86), we obtain that

$$\begin{aligned}
\Phi &= D\boldsymbol{\phi}(\mathbf{0})\mathbf{T}_E\boldsymbol{\Xi} + \mathcal{O}(|\boldsymbol{H}|) + \mathcal{O}\left(|(\boldsymbol{\Xi},\boldsymbol{H})|^{1+\beta}\right), \\
\hat{\Phi} &= D\boldsymbol{\phi}(\mathbf{0})\mathbf{T}_E e^{\boldsymbol{\Lambda}_E\Delta t}\boldsymbol{\Xi} + \mathcal{O}(|\boldsymbol{H}|) + \mathcal{O}\left(|(\boldsymbol{\Xi},\boldsymbol{H})|^{1+\beta}\right),
\end{aligned} \tag{87}$$

where we have used the boundedness of the sampling time Δt and the near-identity nature of the linearizing mapping (82), which implies

$$\mathcal{O}\left(|\boldsymbol{\Xi}|^\beta\right) = \mathcal{O}\left(|\mathbf{P}_E\mathbf{X}|^\beta\right), \qquad \mathcal{O}\left(|\boldsymbol{H}|^\beta\right) = \mathcal{O}\left(|\mathbf{P}_F\mathbf{X}|^\beta\right). \tag{88}$$

From the equations (87), we obtain

$$\begin{aligned}
\hat{\boldsymbol{\Phi}}\boldsymbol{\Phi}^{\mathrm{T}} &= \left[D\boldsymbol{\phi}(0)\mathbf{T}_E e^{\boldsymbol{\Lambda}_E\Delta t}\boldsymbol{\Xi} + \mathcal{O}(|\boldsymbol{H}|) + \mathcal{O}\left(|(\boldsymbol{\Xi},\boldsymbol{H})|^{1+\beta}\right)\right]\left[D\boldsymbol{\phi}(0)\mathbf{T}_E\boldsymbol{\Xi} + \mathcal{O}(|\boldsymbol{H}|) + \mathcal{O}\left(|(\boldsymbol{\Xi},\boldsymbol{H})|^{1+\beta}\right)\right]^{\mathrm{T}} \\
&= D\boldsymbol{\phi}(0)\mathbf{T}_E e^{\boldsymbol{\Lambda}_E\Delta t}\boldsymbol{\Xi}\boldsymbol{\Xi}^{\mathrm{T}}\mathbf{T}_E^{\mathrm{T}}\left[D\boldsymbol{\phi}(\mathbf{0})\right]^{\mathrm{T}} + \mathcal{O}\left(|\boldsymbol{H}|^2, |\boldsymbol{H}|\,|(\boldsymbol{\Xi},\boldsymbol{H})|^{1+\beta}, |(\boldsymbol{\Xi},\boldsymbol{H})|^{2+2\beta}\right), \\
\left(\boldsymbol{\Phi}\boldsymbol{\Phi}^{\mathrm{T}}\right)^{\dagger} &= \left\{\left[D\boldsymbol{\phi}(\mathbf{0})\mathbf{T}_E\boldsymbol{\Xi} + \mathcal{O}(|\boldsymbol{H}|) + \mathcal{O}\left(|(\boldsymbol{\Xi},\boldsymbol{H})|^{1+\beta}\right)\right]\left[D\boldsymbol{\phi}(\mathbf{0})\mathbf{T}_E\boldsymbol{\Xi} + \mathcal{O}(|\boldsymbol{H}|) + \mathcal{O}\left(|(\boldsymbol{\Xi},\boldsymbol{H})|^{1+\beta}\right)\right]^{\mathrm{T}}\right\}^{\dagger} \\
&= \left[D\boldsymbol{\phi}(\mathbf{0})\mathbf{T}_E\boldsymbol{\Xi}\boldsymbol{\Xi}^{\mathrm{T}}\mathbf{T}_E^{\mathrm{T}}\left[D\boldsymbol{\phi}(\mathbf{0})\right]^{\mathrm{T}} + \mathcal{O}\left(|\boldsymbol{H}|^2, |\boldsymbol{H}|\,|(\boldsymbol{\Xi},\boldsymbol{H})|^{1+\beta}, |(\boldsymbol{\Xi},\boldsymbol{H})|^{2+2\beta}\right)\right]^{\dagger}.
\end{aligned} \tag{89}$$

By the first assumption in (A4) of the theorem, $\boldsymbol{\Phi}\boldsymbol{\Phi}^{\mathrm{T}}$ is invertible, and hence $\left(\boldsymbol{\Phi}\boldsymbol{\Phi}^{\mathrm{T}}\right)^{\dagger} = \left(\boldsymbol{\Phi}\boldsymbol{\Phi}^{\mathrm{T}}\right)^{-1}$. Therefore, by the second equation in (89), for small enough $|\boldsymbol{\Xi}|$ and $|\boldsymbol{H}|$, the matrix $D\boldsymbol{\phi}(\mathbf{0})\mathbf{T}_E\boldsymbol{\Xi}\boldsymbol{\Xi}^{\mathrm{T}}\mathbf{T}_E^{\mathrm{T}}\left[D\boldsymbol{\phi}(\mathbf{0})\right]^{\mathrm{T}}$ is also invertible and

$$\left(\boldsymbol{\Phi}\boldsymbol{\Phi}^{\mathrm{T}}\right)^{\dagger} = \left[D\boldsymbol{\phi}(\mathbf{0})\mathbf{T}_E\boldsymbol{\Xi}\boldsymbol{\Xi}^{\mathrm{T}}\mathbf{T}_E^{\mathrm{T}}\left[D\boldsymbol{\phi}(\mathbf{0})\right]^{\mathrm{T}}\right]^{-1} + \mathcal{O}\left(|\boldsymbol{H}|^2, |\boldsymbol{H}|\,|(\boldsymbol{\Xi},\boldsymbol{H})|^{1+\beta}, |(\boldsymbol{\Xi},\boldsymbol{H})|^{2+2\beta}\right). \tag{90}$$

Then, by assumption (A3) of the theorem, we can write

$$\left(\boldsymbol{\Phi}\boldsymbol{\Phi}^{\mathrm{T}}\right)^{\dagger} = \left(\mathbf{T}_E^{\mathrm{T}}\left[D\boldsymbol{\phi}(\mathbf{0})\right]^{\mathrm{T}}\right)^{-1}\left(\boldsymbol{\Xi}\boldsymbol{\Xi}^{\mathrm{T}}\right)^{-1}\left(D\boldsymbol{\phi}(\mathbf{0})\mathbf{T}_E\right)^{-1}+\mathcal{O}\left(\left|\boldsymbol{H}\right|^2,\left|\boldsymbol{H}\right|\left|(\boldsymbol{\Xi},\boldsymbol{H})\right|^{1+\beta},\left|(\boldsymbol{\Xi},\boldsymbol{H})\right|^{2+2\beta}\right). \tag{91}$$

Substitution of formulas (90)-(91) into eq. (8) gives

$$\begin{aligned}
\boldsymbol{\mathcal{D}} &= \hat{\boldsymbol{\Phi}}\boldsymbol{\Phi}^{\mathrm{T}}\left(\boldsymbol{\Phi}\boldsymbol{\Phi}^{\mathrm{T}}\right)^{\dagger} = \hat{\boldsymbol{\Phi}}\boldsymbol{\Phi}^{\mathrm{T}}\left(\boldsymbol{\Phi}\boldsymbol{\Phi}^{\mathrm{T}}\right)^{-1} \\
&= \left[D\boldsymbol{\phi}(0)\mathbf{T}_E e^{\boldsymbol{\Lambda}_E\Delta t}\boldsymbol{\Xi}\boldsymbol{\Xi}^{\mathrm{T}}\mathbf{T}_E^{\mathrm{T}}\left[D\boldsymbol{\phi}(\mathbf{0})\right]^{\mathrm{T}}+\mathcal{O}\left(\left|\boldsymbol{H}\right|^2,\left|\boldsymbol{H}\right|\left|(\boldsymbol{\Xi},\boldsymbol{H})\right|^{1+\beta},\left|(\boldsymbol{\Xi},\boldsymbol{H})\right|^{2+2\beta}\right)\right] \\
&\times\left[\left(\mathbf{T}_E^{\mathrm{T}}\left[D\boldsymbol{\phi}(\mathbf{0})\right]^{\mathrm{T}}\right)^{-1}\left(\boldsymbol{\Xi}\boldsymbol{\Xi}^{\mathrm{T}}\right)^{-1}\left(D\boldsymbol{\phi}(\mathbf{0})\mathbf{T}_E\right)^{-1}+\mathcal{O}\left(\left|\boldsymbol{H}\right|^2,\left|\boldsymbol{H}\right|\left|(\boldsymbol{\Xi},\boldsymbol{H})\right|^{1+\beta},\left|(\boldsymbol{\Xi},\boldsymbol{H})\right|^{2+2\beta}\right)\right] \\
&= D\boldsymbol{\phi}(0)\mathbf{T}_E e^{\boldsymbol{\Lambda}_E\Delta t}\left(D\boldsymbol{\phi}(\mathbf{0})\mathbf{T}_E\right)^{-1}+\mathcal{O}\left(\left|\boldsymbol{\Xi}\right|^{-2}\left|\boldsymbol{H}\right|^2,\left|\boldsymbol{\Xi}\right|^{-2}\left|\boldsymbol{H}\right|\left|(\boldsymbol{\Xi},\boldsymbol{H})\right|^{1+\beta},\left|\boldsymbol{\Xi}\right|^{-2}\left|(\boldsymbol{\Xi},\boldsymbol{H})\right|^{2+2\beta}\right) \\
&= D\boldsymbol{\phi}(0)\mathbf{T}_E e^{\boldsymbol{\Lambda}_E\Delta t}\left(D\boldsymbol{\phi}(\mathbf{0})\mathbf{T}_E\right)^{-1}+\mathcal{O}\left(\left|\boldsymbol{\Xi}\right|^{\beta}\right),
\end{aligned}$$

where we have use the second assumption in (A4) of the Theorem. This completes the proof of Theorem 1, given the order-of-magnitude relations (88).

Remark 4. In systems where the slowest decaying modes are oscillatory, the slow spectral subspace E is always even dimensional, as it is spanned by the real and imaginary parts of the generalized eigenvectors of $\mathbf{A}$ that correspond to complex conjugate pairs of eigenvalues. For such predominantly oscillatory systems, therefore, the number d of observables used in DMD has to be an even number for assumption (A3) to hold.

Remark 5. In most applications the dynamical system will have higher degree of smoothness, i.e., we will have $\mathbf{f} \in C^r$ for some $r \in \mathbb{N}^+ \cup \{\infty, a\}$, with C^a referring to the space of analytic functions. Then, under further nonresonance conditions of the spectrum of $\mathbf{A}$, the spectral submanifold $\mathcal{W}(E)$ will also be of class C^r, as we discuss in Section 4. In those cases, for the approximate topological equivalence holds in the statement of Theorem 1 hold on the full domain of attraction of the $\mathbf{x} = \mathbf{0}$ fixed point within $\mathcal{W}(E)$, as one deduces from the linearization-in-the-large results of Lan and Mezic [35] and Kvalheim and Revzen [34].[5] In Theorem 1, our objective was to give a minimal set of conditions under which DMD can be justified as an approximate, leading-order, d-dimensional reduced model for the original nonlinear system. These minimal conditions only require hyperbolicity, i.e., robustness of the spectrum under perturbations, without insisting on the lack of resonances in the spectrum. This is advantageous in data-driven applications in which details of the spectrum are generally not known.

Remark 6. As in the case of finite-dimensional systems, linearization theorems guaranteeing higher-degree of smoothness for the linearizing transformations are also available in infinite dimensions, but these require various non-resonance conditions on the spectrum of $\mathbf{A}$ (see, e.g., Elbialy [16]). In Theorem 3, as in the finite-dimensional case, our objective was to present a minimal set of assumptions under which DMD can be justified without the detailed knowledge of the spectrum of $\mathbf{A}$.

Remark 7. We note that the more general Theorem 1.5 of Newhouse [41] also shows $C^{1,\alpha}$ linearizability for a class of systems that have so called $\alpha-$*hyperbolic* (as opposed to stable hyperbolic) fixed points. This would enable us to waive the requirement in Theorem 3 that $\mathbf{A}$ is a contraction and also allow for expanding directions within the spectral subspace E, as we did in our remarks after Theorems 1 and 2. The defining properties of these α-hyperbolic systems are, however, complicated to verify and do not hold for general hyperbolic fixed points even in finite-dimensional Banach spaces.

[5] Note that these global linearization results rely on the existence of a C^1 local linearization, which generally only exists for class C^2 dynamical systems. Consequently, one cannot use the available global linearization results within $\mathcal{W}(E)$ under the general assumptions of Theorem 1, which only guarantee C^1 differentiability for the reduced flow in $\mathcal{W}(E)$.

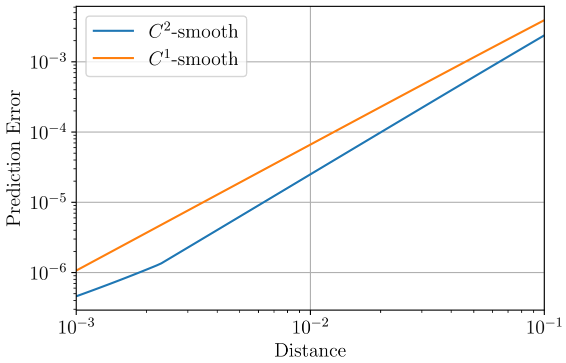

Figure 10: Prediction accuracy of DMD on C^1- and C^2-smooth systems (92). The orange curve shows the accuracy of DMD for $\alpha = 0.9$, while the blue curve corresponds to $\alpha = 1$.

For instance, the simple three-dimensional example of Hartman [23] (eq. (8) in that reference) of a non-C^1 linearizable system has a hyperbolic fixed point that is not α-hyperbolic in the sense of Newhouse [41].

C Necessity of assumptions (A2)-(A4) of Theorem 1

Assumption (A2) of Theorem 1 requires C^2 smoothness for the dynamical system (11). We now show on an example that if only C^1 smoothness holds for the dynamical system, then the DMD approximation can be less accurate. Consider the 1D system

$$\dot{x} = -x + x^{1+\alpha}. \tag{92}$$

The origin $x = 0$ is an asymptotically stable fixed point. For $\alpha > 0$, the system is C^1 at the origin, but the second derivative $\frac{\partial^2 \dot{x}}{\partial x^2} = \alpha^2 x^{\alpha-1}$ is singular at $x = 0$ for $\alpha < 1$. We compare the accuracy of a simple DMD prediction for $\alpha = 1$, which corresponds to C^2 smoothness and for $\alpha = 0.9$, which corresponds to only C^1-smoothness at $x = 0$. The DMD prediction errors can be seen in Fig. 10 as a function of the initial distance of the trajectory from the origin. Although the C^1-smooth system is close to the C^2system, a large difference can be observed in the prediction accuracy of DMD asymptotically as $x \to 0$.

Assumption (A3) of Theorem 1 requires a specific nondegeneracy condition to hold for the observable in order for DMD to give a meaningful approximation. We now illustrate on an example what happens if this nondegeneracy condition is not satisfied. Consider the chain of nonlinear oscillators in Section 5.5. Let us denote the normal modes of the mechanical system (66) linearized around the stable fixed point $\mathbf{q} = \mathbf{0}$, as $\hat{\mathbf{q}}_j(t) = \hat{\mathbf{q}}_j(0)e^{\lambda_j t}$, where the mode shapes $\hat{\mathbf{q}}_j(0) \in \mathbb{R}^5$ satisfy

$$\left(\lambda_j^2 \mathbf{M} + \lambda_j \mathbf{C} + \mathbf{K}\right) \hat{\mathbf{q}}_j(0) = 0.$$

Therefore, in the phase space spanned by the variable

$$\mathbf{x} = (q_1, q_2, ..., q_5, \dot{q}_1, \ldots, \dot{q}_5) \in \mathbb{R}^{10},$$

the eigenvectors of the linear part of (66)are given as

$$\mathbf{e}_j = \left(\hat{\mathbf{q}}_j(0), \lambda_j \hat{\mathbf{q}}_j(0)\right)^T.$$

With our choice of parameters, the slowest pair of eigenvalues are

$$\lambda_{1,2} = -0.0012 \pm i0.2825,$$

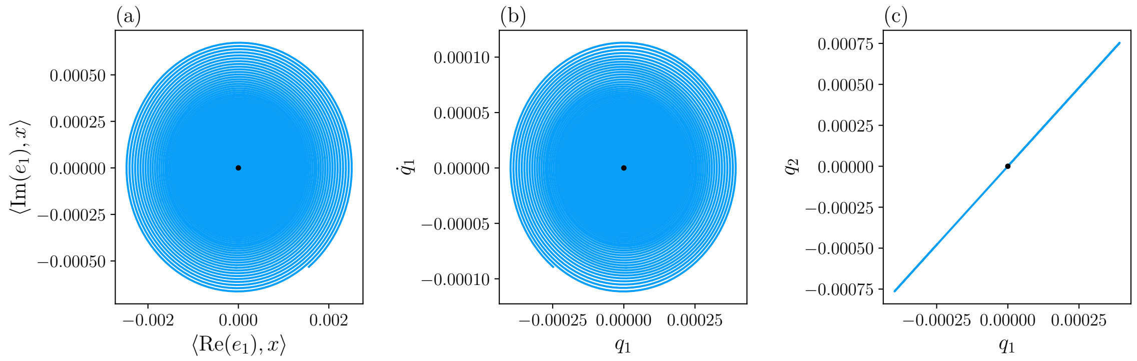

Figure 11: The dynamics near the $\mathbf{x} = \mathbf{0}$ point on the slow 2D SSM $\mathcal{W}(E)$ of system (66) represented via different observables (a) Observable $\boldsymbol{\phi}_1(\mathbf{x})$ (b) Observable $\boldsymbol{\phi}_2(\mathbf{x})$ (c) Observable $\boldsymbol{\phi}_3(\mathbf{x})$.

and hence there exists a 2D slow SSM tangent to the slow spectral subspace

$$E = \operatorname{span}\left([\operatorname{Re}(\mathbf{e}_1), \operatorname{Im}(\mathbf{e}_1)]\right) = \operatorname{span}\left(\left[\hat{\mathbf{q}}_1(0), \operatorname{Re}(\lambda_1)\,\hat{\mathbf{q}}_1(0)\right], \left[0, \operatorname{Im}(\lambda_1)\,\hat{\mathbf{q}}_1(0)\right]\right),$$

where $\mathbf{e}_1$ is the eigenvector corresponding to λ_1.

As the dimension of $\mathcal{W}(E)$ is $d = 2$, we consider three possible 2D observables with respect to which Theorem 1 could potentially be applied:

$$\boldsymbol{\phi}_1(\mathbf{x}) = \begin{pmatrix} \langle \operatorname{Re}(\mathbf{e}_1), \mathbf{x}\rangle \\ \langle \operatorname{Im}(\mathbf{e}_1), \mathbf{x}\rangle \end{pmatrix} \quad \boldsymbol{\phi}_2(\mathbf{x}) = \begin{pmatrix} q_1 \\ \dot{q}_1 \end{pmatrix} \quad \boldsymbol{\phi}_3(\mathbf{x}) = \begin{pmatrix} q_1 \\ q_2 \end{pmatrix}.$$

The Jacobians of these observables with respect to x can be computed as

$$D\boldsymbol{\phi}_1(\mathbf{0}) = \left(\operatorname{Re}(\mathbf{e}_1)\,\operatorname{Im}(\mathbf{e}_1)\right)^{\mathrm{T}} = \begin{pmatrix} * & * & * & \cdots & * \\ 0 & 0 & 0 & \cdots & * \end{pmatrix},$$
$$D\boldsymbol{\phi}_2(\mathbf{0}) = \begin{pmatrix} 1 & 0 & 0 & \cdots & 0 \\ 0 & 1 & 0 & \cdots & 0 \end{pmatrix},$$
$$D\boldsymbol{\phi}_3(\mathbf{0}) = \begin{pmatrix} 1 & 0 & 0 & \cdots & 0 \\ 0 & 0 & 1 & \cdots & 0 \end{pmatrix},$$

where $*$ denotes nonzero entries. Therefore, we require

$$\operatorname{rank}\left[D\boldsymbol{\phi}_{1,2,3}(\mathbf{0})\,|_E\right] = 2. \tag{93}$$

The restriction of the Jacobians of the observables to E is $D\boldsymbol{\phi}_i(\mathbf{0})[\operatorname{Re}(\mathbf{e}_1), \operatorname{Im}(\mathbf{e}_1)]$. It can be seen from the structure of the matrices, that for $\boldsymbol{\phi}_1$ and $\boldsymbol{\phi}_2$, the rank of the restricted Jacobian is 2, but for $\boldsymbol{\phi}_3$, it is only of rank 1. Therefore, the observable vector $\boldsymbol{\phi}_3$ cannot be used to parametrize the SSM and to derive a reduced-order model.

Figures 11a-b show that observing the dynamics via $\boldsymbol{\phi}_1(x)$ and $\boldsymbol{\phi}_2(x)$ indeed results in a successful embedding of the dynamics on $\mathcal{W}(E)$ near the fixed point. At the same time, Fig. 11c shows that using the observable $\boldsymbol{\phi}_3(x)$ does not correctly reproduce the dynamics near the origin.

Assumption (A4) of Theorem 1 states that the collected data has to be rich enough to a neighborhood of the SSM near the fixed point. To illustrate this, we modify the linear system in (59) to have three real eigenvalues by letting $\boldsymbol{\Lambda} = \operatorname{diag}(a, b, c) \in \mathbb{R}^3$. We then collect the resulting trajectories in the phase space in the observable data matrix

$$\boldsymbol{\Phi} = \begin{pmatrix} x_1(0) & ax_1(0) & & a^n x_1(0) \\ x_2(0) & bx_2(0) & \cdots & b^n x_2(0) \\ x_3(0) & cx_3(0) & & c^n x_3(0) \end{pmatrix}.$$

Assuming $c < b < a < 0$, we seek to identify from the data matrix Φ the 2D slow eigenspace $\mathcal{W}(E) = E$ corresponding to the eigenvalues a and b. Assume now that $x_2(0) = x_3(0) = 0$ holds. In this case, the second and third rows of Φ are identically zero and hence its rank is only 1, which violates assumption (A4) of Theorem 1. Indeed, the trajectory observed in $\mathbf{\Phi}$ is restricted to the slowest 1D eigenspace $\mathcal{W}(E_1) = E_1$ corresponding to the eigenvalue a and hence cannot be used to approximate the shape and internal dynamics of $\mathcal{W}(E)$.

In practice, a generic trajectory of a nonlinear system would not be restricted to a lower-dimensional invariant subspace and hence the data matrix Φ would have full rank with probability one. However, if the available data is close to a lower-dimensional invariant subspace, the condition number of the data matrix may become prohibitively large. So a near-failure of assumption (A4) is already detrimental to the quality of the results obtained from DMD.

D Proof of Theorem 4

For $\mathbf{f}_2 \in C^r$ and under the nonresonance conditions

$$\lambda_k \neq \sum_{j=1}^{n} m_j \lambda_j, \qquad j,k = 1,\dots,n, \qquad \sum_{j=1}^{n} m_j \geq 2, \tag{94}$$

the C^∞ linearization theorems of Sternberg [52] and Poincaré [46] guarantee the local existence of a near-identity, linearizing change of coordinates of the form

$$\mathbf{x} = \mathbf{y} + \mathbf{h}(\mathbf{y}), \qquad \mathbf{y} \in U \subset \mathbb{R}^n, \qquad \mathbf{h}(\mathbf{y}), \mathbf{h}^{-1}(\mathbf{y}) = \mathcal{O}\left(|\mathbf{y}|^2\right), \tag{95}$$

with $\mathbf{h} \in C^r$. Additionally, a strengthening of Sternberg's linearization theorem by Kvalheim and Revzen [34][6] implies that the transformation in eq. (95) is unique and globally defined on the full domain of attraction of the fixed point at the origin. As pointed out by Haller et al. [22], this implies the existence of a unique d-dimensional, normally attracting spectral submanifold $\mathcal{W}(E) \in C^\infty$ for system (11) whose internal dynamics govern the longer-term behavior of all trajectories near the origin.[7]

By the main assumption of the theorem, we have $\mathrm{Re}\lambda_1 < 0$. Under this assumption, the infinitely many nonresonance conditions in (94) simplify to finitely many. Indeed, if we define the spectral quotient as the positive integer Q given by

$$Q = \left\lfloor \frac{\max_i |\mathrm{Re}\,\lambda_i|}{\min_i |\mathrm{Re}\,\lambda_i|} \right\rfloor + 1,$$

Then, for all $m_j \in \mathbb{N}$ with $\sum_{j=1}^{n} m_j > Q$, we have the estimate

$$\begin{aligned}\sum_{j=1}^{n} m_j \mathrm{Re}\,\lambda_j &\leq -\sum_{j=1}^{n} m_j \min_i |\mathrm{Re}\,\lambda_i| < -\sum_{j=1}^{n} m_j \frac{\max_i |\mathrm{Re}\,\lambda_i|}{Q} = -\max_i |\mathrm{Re}\,\lambda_i| \frac{\sum_{j=1}^{n} m_j}{Q} \\ &< -\max_i |\mathrm{Re}\,\lambda_i|,\end{aligned}$$

implying

$$\sum_{j=1}^{n} m_j \mathrm{Re}\,\lambda_j < \mathrm{Re}\,\lambda_k, \qquad k = 1,\dots,n, \quad \sum_{j=1}^{n} m_j > Q. \tag{96}$$

[6] This generalizes the results of Lan and Mezic [35] in the setting of the C^1 linearization theorem by Hartman [23].

[7] Cabré et al. [10] guarantees the existence and uniqueness of $\mathcal{W}(E) \in C^\infty$ under weaker nonresonance conditions that allow resonances inside E but no resonances of the form (94) with $\lambda_k \in \mathrm{Spect}\,(A|_F)$ and $\lambda_j \in \mathrm{Spect}\,(\mathbf{A}|_E)$. We do not rely on these results here, as the next step in our construction is local linearization within $\mathcal{W}(E)$, which cannot be carried out in the presence of resonances inside E.

Consequently, in this case, no integer resonance among the eigenvalues of $\mathbf{A}$ can occur for $\sum_{j=1}^{n} m_j > Q$ and hence the nonresonance condition (94) can be relaxed to assumption (A1) of Theorem 4.

Based on these results, we apply the C^r linearizing transformation (95) followed by a linear change of coordinates to $(\boldsymbol{\xi}, \boldsymbol{\eta})$uses in the proof of Theorem 1 (see eq. (82)). As se have seen there, this coordinate change maps the spectral submanifold $\mathcal{W}(E)$ into the $\boldsymbol{\eta} = \mathbf{0}$ plane. On $\mathcal{W}(E)$, the reduced dynamics obeys the linear ODE

$$\dot{\boldsymbol{\xi}} = \boldsymbol{\Lambda}_E \boldsymbol{\xi}, \qquad \boldsymbol{\Lambda}_E = \left(\mathbf{T}^{-1}\mathbf{A}\mathbf{T}\right)|_E, \tag{97}$$

where $\boldsymbol{\Lambda}_E$, the real Jordan canonical form of $\mathbf{A}|_E$, has eigenvalues $\lambda_1, \ldots, \lambda_d$.

Restricted to $\mathcal{W}(E)$, we can express the d-dimensional observable vector $\boldsymbol{\phi}(\mathbf{x})$ as

$$\boldsymbol{\varphi}(\boldsymbol{\xi}) = \boldsymbol{\phi}\left(\mathbf{T}\begin{pmatrix}\boldsymbol{\xi}\\ \mathbf{0}\end{pmatrix} + \mathbf{h}\begin{pmatrix}\boldsymbol{\xi}\\ \mathbf{0}\end{pmatrix}\right) \in \mathbb{R}^d. \tag{98}$$

On the manifold $\mathcal{W}(E)$, we can use $\boldsymbol{\varphi}$ locally near the origin as a new variable if the coordinate change from $\boldsymbol{\xi}$ to $\boldsymbol{\varphi}$ is a C^r diffeomorphism on $\mathcal{W}(E)$, i.e., $D_{\boldsymbol{\xi}}\boldsymbol{\varphi}$ is nonsingular at the origin. To see if this is the case, note that

$$D_{\boldsymbol{\xi}}\boldsymbol{\psi}(\boldsymbol{\xi}) = D\boldsymbol{\phi}\left(\mathbf{T}\begin{pmatrix}\boldsymbol{\xi}\\ \mathbf{0}\end{pmatrix}\right)\mathbf{T}\begin{pmatrix}\mathbf{I}_{d\times d}\\ \mathbf{0}\end{pmatrix},$$

which gives

$$D_{\boldsymbol{\xi}}\boldsymbol{\gamma}(\mathbf{0}) = D\boldsymbol{\phi}\left(\mathbf{0}\right)\mathbf{T}\begin{pmatrix}\mathbf{I}_{d\times d}\\ \mathbf{0}\end{pmatrix} = D\boldsymbol{\phi}\left(\mathbf{0}\right)\mathbf{T}_E,$$

with the operator $\mathbf{T}_E$ defined in (15). By assumption (35) of the theorem, we have

$$\det\left[D\boldsymbol{\phi}\left(\mathbf{0}\right)\mathbf{T}_E\right] \neq 0, \tag{99}$$

and hence, by the inverse function theorem, $\boldsymbol{\xi}$ can be expressed from the relationship (98) near the origin as a smooth function of $\boldsymbol{\varphi}$. Specifically, there exists a C^r diffeomorphism $\boldsymbol{\psi}\colon \mathbb{R}^d \to \mathbb{R}^d$ such that

$$\boldsymbol{\xi} = \boldsymbol{\psi}(\boldsymbol{\varphi}), \qquad \boldsymbol{\psi}(\mathbf{0}) = \mathbf{0}, \quad D_{\boldsymbol{\varphi}}\boldsymbol{\psi}(\boldsymbol{\varphi}) = \left[D_{\boldsymbol{\xi}}\boldsymbol{\varphi}(\boldsymbol{\xi})\right]^{-1}. \tag{100}$$

Differentiating the first equation in (100), substituting eq. (97), using the relations (100) and Taylor expanding the result around $\boldsymbol{\varphi} = \mathbf{0}$, we obtain the reduced dynamics on $\mathcal{W}(E)$ in the form

$$\begin{aligned}\dot{\boldsymbol{\varphi}} &= \left[D_{\boldsymbol{\varphi}}\boldsymbol{\psi}(\boldsymbol{\varphi})\right]^{-1}\boldsymbol{\Lambda}_E\boldsymbol{\psi}(\boldsymbol{\varphi})\\ &= D_{\boldsymbol{\xi}}\boldsymbol{\varphi}(\mathbf{0})\boldsymbol{\Lambda}_E\left[D_{\boldsymbol{\xi}}\boldsymbol{\varphi}(\mathbf{0})\right]^{-1}\boldsymbol{\varphi} + \mathbf{q}\left(\boldsymbol{\varphi}\right), \qquad \mathbf{q}\left(\boldsymbol{\varphi}\right) = o\left(|\boldsymbol{\varphi}|\right)\end{aligned} \tag{101}$$

for some C^r function $\mathbf{q}\colon U \subset \mathbb{R}^d \to \mathbb{R}^d$ defined in a neighborhood of $\boldsymbol{\varphi} = \mathbf{0}$. This proves statement (i) of the theorem.

We have seen that $D_{\boldsymbol{\xi}}\boldsymbol{\varphi}(\mathbf{0})$ is nonsingular and hence the linear part of the nonlinear ODE in (101) has the same eigenvalues as $\boldsymbol{\Lambda}_E$ does. $\left(\mathbf{T}^{-1}\mathbf{A}\mathbf{T}\right)|_E$. Consequently, the origin of system (102) is a hyperbolic fixed point. If the nonresonance conditions (34) hold for the full spectrum of $\mathbf{A}$, then they also hold for any subset of this spectrum, i.e., for the eigenvalues of $\boldsymbol{\Lambda}_E$ as well. Consequently, from the linearization theorems of Sternberg [52] and Kvalheim and Revzen [34], we can also deduce a similar near-identity, C^r linearizing diffeomorphism

$$\boldsymbol{\varphi} = \boldsymbol{\gamma} + \boldsymbol{\ell}(\boldsymbol{\gamma}), \qquad \boldsymbol{\ell}(\boldsymbol{\gamma}) = o\left(|\boldsymbol{\gamma}|\right), \quad \boldsymbol{\ell} \in C^r,$$

for $r \in \mathbb{N}^+ \cup \{\infty, a\}$ under the nonresonance assumption (34). In the new $\boldsymbol{\gamma}$ coordinates, the dynamics on the SSM $\mathcal{W}\left(E\right)$ is governed by the linear ODE

$$\dot{\boldsymbol{\gamma}} = D_{\boldsymbol{\xi}}\boldsymbol{\gamma}(\mathbf{0})\boldsymbol{\Lambda}_E\left[D_{\boldsymbol{\xi}}\boldsymbol{\gamma}(\mathbf{0})\right]^{-1}\boldsymbol{\gamma}, \tag{102}$$

which proves statement (ii) of the theorem.

The partial differential equation (39) can then be obtained by differentiating eq. (37) and substituting eqs. (36) and (38) into the resulting equation. The expansions (40) and (41) then follow from the guaranteed smoothness class of the linearizing transformation for general r and for $r = a$, respectively. This concludes the proof of statement (iii) of the theorem.

E Reduced dynamics on periodically forced SSMs

Let us denote the time-T map for the periodically forced system by $\mathbf{P}_\varepsilon^T$. Since $\mathbf{P}_0^T$ represents the period-T sampling of the flow map of the unperturbed system, it has a fixed point at $\mathbf{x} = \mathbf{0}$. By the implicit function theorem, $\mathbf{P}_\varepsilon^T$ also has a fixed point $O(\varepsilon)-$close to $\mathbf{x} = \mathbf{0}$. This corresponds to a periodic orbit of (52) close to the origin. As explained by Haller et al. [22], the C^∞ linearization results of Sternberg [52] and Poincaré [46] also establish the smoothness of the linearizations in ε. Applied to the discrete dynamical system defined by the Poincaré-map, these results guarantee the existence of a linearizing transformation of the form

$$\mathbf{x} = \mathbf{y} + \mathbf{h}^\varepsilon(\mathbf{y}), \qquad \mathbf{y} \in U \subset \mathbb{R}^n, \qquad \mathbf{h}(\mathbf{y}), \mathbf{h}^{-1}(\mathbf{y}) = \mathcal{O}\left(|\mathbf{y}|^2, \varepsilon|\mathbf{y}|^2\right),$$

with $\mathbf{h} \in C^r$ in both $\mathbf{y}$ and ε.

We now apply the ε-dependent linearizing transformation followed by the linear change of coordinates to the coordinates $(\boldsymbol{\xi}, \boldsymbol{\eta})$ introduced in the proof of Theorem 4. Specifically, let

$$\mathbf{x} = \mathbf{T}\mathbf{y} + \mathbf{h}^\varepsilon(\mathbf{T}\mathbf{y}), \quad \mathbf{y} = (\boldsymbol{\xi}, \boldsymbol{\eta})^{\mathrm{T}} \in \mathbb{R}^d \times \mathbb{R}^{n-d}, \tag{103}$$

with the matrix $\mathbf{T}$ defined in formula (15), i.e., containing eigenvalues of $\mathbf{A}$. As the change of coordinates (103) is smooth in ε, it can be written as $\mathbf{x} = \mathbf{T}\mathbf{y} + \mathbf{h}^0(\mathbf{T}\mathbf{y}) + O(|y|^2\varepsilon)$. Differentiation of (103) with respect to time yields

$$\dot{\mathbf{x}} = (\mathbf{T} + D\mathbf{h}^\varepsilon(\mathbf{T}\mathbf{y})\mathbf{T})\,\dot{\mathbf{y}} = \mathbf{A}(\mathbf{T}\mathbf{y} + \mathbf{h}^\varepsilon(\mathbf{T}\mathbf{y})) + \tilde{\mathbf{f}}(\mathbf{T}\mathbf{y} + \mathbf{h}^\varepsilon(\mathbf{T}\mathbf{y})) + \varepsilon\mathbf{F}(\mathbf{x} + h^\varepsilon(\mathbf{T}\mathbf{y}), t),$$

which can be rearranged as

$$\begin{aligned}\dot{\mathbf{y}} &= \left(\mathbf{T} + D\mathbf{h}^0(\mathbf{T}\mathbf{y})\mathbf{T} + O(|\mathbf{y}|\varepsilon)\right)^{-1}\left[\mathbf{A}\left(\mathbf{T}\mathbf{y} + \mathbf{h}^0(\mathbf{T}\mathbf{y}) + O(|\mathbf{y}|^2\varepsilon)\right)\right] \\ &\quad + \left(\mathbf{T} + D\mathbf{h}^0(\mathbf{T}\mathbf{y})\mathbf{T} + O(|\mathbf{y}|\varepsilon)\right)^{-1}\left[\tilde{\mathbf{f}}(\mathbf{T}\mathbf{y} + \mathbf{h}^0(\mathbf{T}\mathbf{y}) + O(|\mathbf{y}|^2\varepsilon)) + \varepsilon\mathbf{F}(\mathbf{T}\mathbf{y} + \mathbf{h}^0(\mathbf{T}\mathbf{y}), t)\right].\end{aligned} \tag{104}$$

Since $y + h^0(y)$ linearizes (52) for $\varepsilon = 0$, (104) simplifies to

$$\dot{\mathbf{y}} = \boldsymbol{\Lambda}\mathbf{y} + \left(\mathbf{T} + D\mathbf{h}^0(\mathbf{T}\mathbf{y})\mathbf{T}\right)^{-1}\varepsilon\mathbf{F}(\mathbf{0}, t) + O(|\mathbf{y}|^2\varepsilon, \mathbf{y}\varepsilon^2), \quad \mathbf{y} = (\boldsymbol{\xi}, \boldsymbol{\eta})^{\mathrm{T}}. \tag{105}$$

The smoothest SSM $\mathcal{W}_\varepsilon(E, t)$ of the periodic orbit is $O(\varepsilon)-$close to that of the fixed point and can be written as

$$\mathcal{W}_\varepsilon(E, t) = \begin{pmatrix} \boldsymbol{\xi} \\ O(\varepsilon) \end{pmatrix}.$$

We now neglect the small, time-dependent correction to the $\boldsymbol{\eta}$-component of $\mathcal{W}_\varepsilon(E, t)$. The reduced dynamics can then be written as the projection of (105) to the $\boldsymbol{\eta} = 0$ plane, which becomes

$$\dot{\boldsymbol{\xi}} = \boldsymbol{\Lambda}_E\boldsymbol{\xi} + \left(\mathbf{T} + D\mathbf{h}^0(\mathbf{T}\mathbf{y})\mathbf{T}\right)^{-1}|_E\varepsilon\mathbf{F}(0, t) + O(|\mathbf{y}|^2\varepsilon, y\varepsilon^2), \qquad \boldsymbol{\Lambda}_E = \left(\mathbf{T}^{-1}\mathbf{A}\mathbf{T}\right)|_E. \tag{106}$$

The restriction of the observable vector $\boldsymbol{\phi}$ to the subspace E can be written as $\boldsymbol{\varphi}(\boldsymbol{\xi})$. Repeating the arguments of D, the change of coordinates $\boldsymbol{\xi} = \boldsymbol{\psi}(\boldsymbol{\varphi})$ can be carried out, which transforms (106) to

$$\dot{\boldsymbol{\varphi}} = D_{\boldsymbol{\xi}}\boldsymbol{\varphi}(\mathbf{0})\boldsymbol{\Lambda}_E\left[D_{\boldsymbol{\xi}}\boldsymbol{\varphi}(\mathbf{0})\right]^{-1}\boldsymbol{\varphi} + \mathbf{q}(\boldsymbol{\varphi}) + \varepsilon\left\{\left[\mathbf{I} + D\mathbf{h}^0\left(D_{\boldsymbol{\xi}}\boldsymbol{\varphi}(\mathbf{0})\right)^{-1}\right]\mathbf{T}_E\right\}^{-1}\mathbf{F}(\mathbf{0}, t) + O(|\boldsymbol{\varphi}|^2\varepsilon, |\boldsymbol{\varphi}|\,\varepsilon^2), \tag{107}$$

$\mathbf{q}(\boldsymbol{\varphi}) = o(|\boldsymbol{\varphi}|)$,

which proves formula (53).

F Forced response from approximate DDL

As stated in Section 4.3.4, the linearizing change of coordinates $\boldsymbol{\varphi} = \boldsymbol{\kappa}(\boldsymbol{\gamma}) = \boldsymbol{\gamma} + \boldsymbol{\ell}(\boldsymbol{\gamma})$ transforms the reduced dynamics of the forced system (107) to the form $\dot{\boldsymbol{\gamma}} = \mathbf{B}\boldsymbol{\gamma} + \varepsilon(\mathbf{I} + D\boldsymbol{\ell}(\boldsymbol{\gamma}))^{-1}\hat{\mathbf{F}}(\mathbf{0}, t)$. Therefore, a trajectory of the forced system may be computed by integrating (55) and transforming to the reduced coordinates $\boldsymbol{\varphi}$ as

$$\boldsymbol{\varphi}(t) = \boldsymbol{\gamma}(t) + \boldsymbol{\ell}(\boldsymbol{\gamma}(t)).$$

An approximate version of (55) may be obtained by assuming (56), i.e., $\varepsilon(\mathbf{I} + D\boldsymbol{\ell}(\boldsymbol{\gamma}))^{-1} = \varepsilon\mathbf{I} + O(\varepsilon|\boldsymbol{\gamma}|) \approx \varepsilon\mathbf{I}$. This results in the forced linear system

$$\dot{\boldsymbol{\gamma}} = \mathbf{B}\boldsymbol{\gamma} + \varepsilon\hat{\mathbf{F}}(\mathbf{0}, t). \tag{108}$$

However, we must keep in mind that the $O(\varepsilon|\gamma|)$ terms are already retained when we compute $\boldsymbol{\varphi}(t) = \boldsymbol{\gamma}(t) + \boldsymbol{\ell}(\boldsymbol{\gamma}(t))$, therefore terms of the same order must also be retained in the dynamics (55). Nevertheless, assumption (56) makes the dynamics (108) an inhomogeneous linear system of ODEs, which is simple to solve. We refer to DDL under assumption (56) as Approximate DDL. We compare forced response predictions of the analytic linearization, DDL, Approximate DDL and DMD on the forced-damped Duffing system discussed in Section 5.3.

In Fig. 12, we repeat the results already presented in Fig. 6, which should now be compared to the forced response predicted using Approximate DDL. Panel (a) shows that although Approximate DDL is accurate only for low amplitudes, it clearly outperforms the DMD-model. For higher amplitudes, predictions are not accurate, since the forced linear system (108), by construction, cannot show nonlinear behavior. In contrast, the analytic linearization and the full DDL continue to yield accurate predictions for the forced response, even in the nonlinear regime.

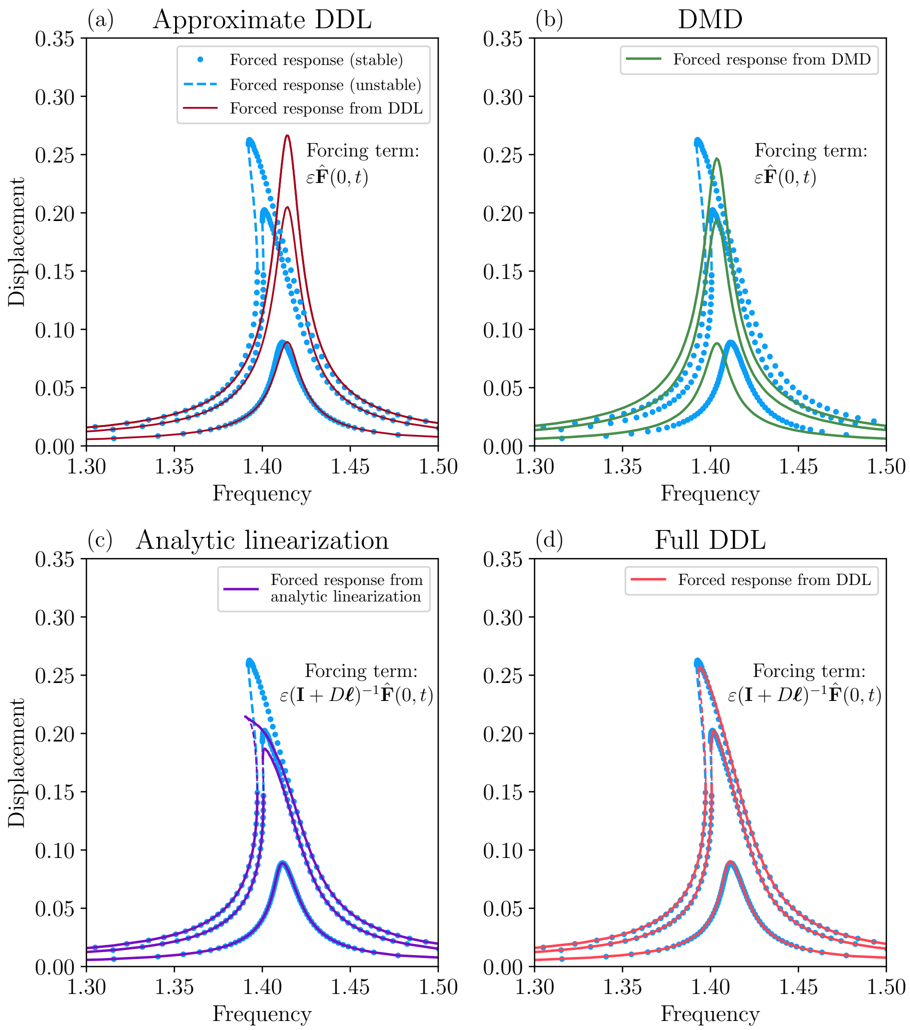

Figure 12: Periodic response of the forced Duffing oscillator (63). Panel (a) shows DDL-predictions under the simplifying assumption (56). Panels (b)-(d) are the same as Fig. 6.
